# Deficiency of myeloid PHD proteins aggravates atherogenesis via macrophage apoptosis and paracrine fibrotic signalling

**DOI:** 10.1093/cvr/cvab152

**Published:** 2021-04-29

**Authors:** Kim van Kuijk, Jasper A F Demandt, Javier Perales-Patón, Thomas L Theelen, Christoph Kuppe, Elke Marsch, Jenny de Bruijn, Han Jin, Marion J Gijbels, Ljubica Matic, Barend M E Mees, Chris P M Reutelingsperger, Ulf Hedin, Erik A L Biessen, Peter Carmeliet, Andrew H Baker, Rafael K Kramann, Leon J Schurgers, Julio Saez-Rodriguez, Judith C Sluimer

**Affiliations:** Cardiovascular Research Institute Maastricht (CARIM), Maastricht University Medical Center (MUMC), Maastricht, Netherlands; Department of Pathology, MUMC, P. Debyelaan 25, 6229HX Maastricht, Netherlands; Institute of Experimental Medicine and Systems Biology, RWTH Aachen University, Aachen, Germany; Cardiovascular Research Institute Maastricht (CARIM), Maastricht University Medical Center (MUMC), Maastricht, Netherlands; Department of Pathology, MUMC, P. Debyelaan 25, 6229HX Maastricht, Netherlands; Institute of Experimental Medicine and Systems Biology, RWTH Aachen University, Aachen, Germany; Institute for Computational Biomedicine, Faculty of Medicine, Heidelberg University, and Heidelberg University Hospital, Bioquant, Heidelberg, Germany; Joint Research Centre for Computational Biomedicine (JRC COMBINE), Faculty of Medicine, RWTH Aachen University, Aachen, Germany; Cardiovascular Research Institute Maastricht (CARIM), Maastricht University Medical Center (MUMC), Maastricht, Netherlands; Department of Pathology, MUMC, P. Debyelaan 25, 6229HX Maastricht, Netherlands; Institute of Experimental Medicine and Systems Biology, RWTH Aachen University, Aachen, Germany; Cardiovascular Research Institute Maastricht (CARIM), Maastricht University Medical Center (MUMC), Maastricht, Netherlands; Department of Pathology, MUMC, P. Debyelaan 25, 6229HX Maastricht, Netherlands; Cardiovascular Research Institute Maastricht (CARIM), Maastricht University Medical Center (MUMC), Maastricht, Netherlands; Department of Pathology, MUMC, P. Debyelaan 25, 6229HX Maastricht, Netherlands; Cardiovascular Research Institute Maastricht (CARIM), Maastricht University Medical Center (MUMC), Maastricht, Netherlands; Department of Pathology, MUMC, P. Debyelaan 25, 6229HX Maastricht, Netherlands; Cardiovascular Research Institute Maastricht (CARIM), Maastricht University Medical Center (MUMC), Maastricht, Netherlands; Department of Pathology, MUMC, P. Debyelaan 25, 6229HX Maastricht, Netherlands; Department of Molecular Genetics, MUMC, Maastricht, Netherlands; Department of Experimental Vascular Biology, Amsterdam UMC, Amsterdam, The Netherlands; GROW-School for Oncology and Developmental Biology, MUMC, Maastricht, Netherlands; Department of Molecular Medicine and Surgery, Karolinska Institute, Stockholm, Sweden; Cardiovascular Research Institute Maastricht (CARIM), Maastricht University Medical Center (MUMC), Maastricht, Netherlands; Department of Vascular Surgery, MUMC, Maastricht, Netherlands; Cardiovascular Research Institute Maastricht (CARIM), Maastricht University Medical Center (MUMC), Maastricht, Netherlands; Department of Biochemistry, MUMC, Maastricht, Netherlands; Department of Molecular Medicine and Surgery, Karolinska Institute, Stockholm, Sweden; Cardiovascular Research Institute Maastricht (CARIM), Maastricht University Medical Center (MUMC), Maastricht, Netherlands; Department of Pathology, MUMC, P. Debyelaan 25, 6229HX Maastricht, Netherlands; Institute for Molecular Cardiovascular Research, RWTH Aachen University, Aachen, Germany; Laboratory of Angiogenesis and Vascular Metabolism, Department of Oncology, KU Leuven, VIB Center for Cancer biology, B-3000 Leuven, Belgium; Cardiovascular Research Institute Maastricht (CARIM), Maastricht University Medical Center (MUMC), Maastricht, Netherlands; BHF Centre for Cardiovascular Sciences (CVS), University of Edinburgh, Edinburgh, UK; Institute of Experimental Medicine and Systems Biology, RWTH Aachen University, Aachen, Germany; Department of Internal Medicine, Nephrology and Transplantation, Erasmus Medical Center, Rotterdam, The Netherlands; Cardiovascular Research Institute Maastricht (CARIM), Maastricht University Medical Center (MUMC), Maastricht, Netherlands; Institute of Experimental Medicine and Systems Biology, RWTH Aachen University, Aachen, Germany; Department of Biochemistry, MUMC, Maastricht, Netherlands; Institute for Computational Biomedicine, Faculty of Medicine, Heidelberg University, and Heidelberg University Hospital, Bioquant, Heidelberg, Germany; Joint Research Centre for Computational Biomedicine (JRC COMBINE), Faculty of Medicine, RWTH Aachen University, Aachen, Germany; Cardiovascular Research Institute Maastricht (CARIM), Maastricht University Medical Center (MUMC), Maastricht, Netherlands; Department of Pathology, MUMC, P. Debyelaan 25, 6229HX Maastricht, Netherlands; BHF Centre for Cardiovascular Sciences (CVS), University of Edinburgh, Edinburgh, UK

**Keywords:** Hypoxia, Fibrosis, Inflammation, Atherosclerosis, Fibroblasts

## Abstract

**Aims:**

Atherosclerotic plaque hypoxia is detrimental for macrophage function. Prolyl hydroxylases (PHDs) initiate cellular hypoxic responses, possibly influencing macrophage function in plaque hypoxia. Thus, we aimed to elucidate the role of myeloid PHDs in atherosclerosis.

**Methods and results:**

Myeloid-specific PHD knockout (PHDko) mice were obtained via bone marrow transplantation (PHD1ko, PHD3ko) or conditional knockdown through lysozyme M-driven Cre recombinase (PHD2cko). Mice were fed high cholesterol diet for 6–12 weeks to induce atherosclerosis. Aortic root plaque size was significantly augmented 2.6-fold in PHD2cko, and 1.4-fold in PHD3ko compared to controls but was unchanged in PHD1ko mice. Macrophage apoptosis was promoted in PHD2cko and PHD3ko mice *in vitro* and *in vivo*, via the hypoxia-inducible factor (HIF) 1α/BNIP3 axis. Bulk and single-cell RNA data of PHD2cko bone marrow-derived macrophages (BMDMs) and plaque macrophages, respectively, showed enhanced HIF1α/BNIP3 signalling, which was validated *in vitro* by siRNA silencing. Human plaque BNIP3 mRNA was positively associated with plaque necrotic core size, suggesting similar pro-apoptotic effects in human. Furthermore, PHD2cko plaques displayed enhanced fibrosis, while macrophage collagen breakdown by matrix metalloproteinases, collagen production, and proliferation were unaltered. Instead, PHD2cko BMDMs enhanced fibroblast collagen secretion in a paracrine manner. *In silico* analysis of macrophage-fibroblast communication predicted SPP1 (osteopontin) signalling as regulator, which was corroborated by enhanced plaque SPP1 protein *in vivo*. Increased SPP1 mRNA expression upon PHD2cko was preferentially observed in foamy plaque macrophages expressing ‘triggering receptor expressed on myeloid cells-2’ (TREM2hi) evidenced by single-cell RNA, but not in neutrophils. This confirmed enhanced fibrotic signalling by PHD2cko macrophages to fibroblasts, *in vitro* as well as *in vivo*.

**Conclusion:**

Myeloid PHD2cko and PHD3ko enhanced atherosclerotic plaque growth and macrophage apoptosis, while PHD2cko macrophages further activated collagen secretion by fibroblasts *in vitro*, likely via paracrine SPP1 signalling through TREM2hi macrophages.

## Graphical Abstract

**Figure:**
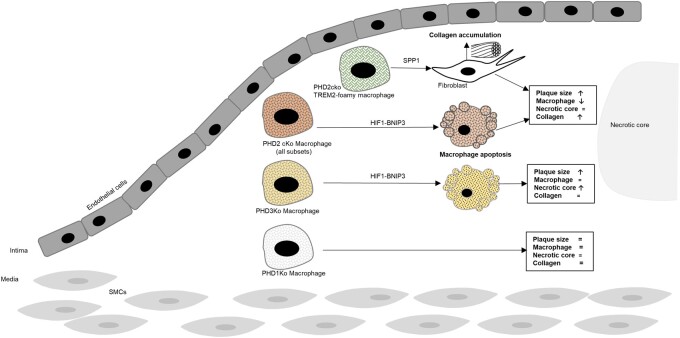

## 1. Introduction

Atherosclerosis is driven by macrophages, which comprise the major myeloid plaque subset.^1^ Macrophages attempt to clear cholesterol and cellular debris accumulated in the intimal wall. However, their function is inhibited as plaque macrophages are hypoxic as result of their high metabolic demand.^2–4^ Restoring murine plaque oxygenation can decrease necrotic plaque content by improving macrophage function.^2^ This indicates that plaque hypoxia is an active participant, rather than an epiphenomenon in atherogenesis.

Three prolyl hydroxylases (PHDs) 1, 2, and 3 are of fundamental importance in hypoxic signalling.^5^ PHDs use oxygen to hydroxylate the transcription factors hypoxia-inducible factor (HIF) 1α and 2α, marking their degradation. Together, PHDs regulate the activity of HIF1α and HIF2α, and downstream mechanisms in order to alleviate detrimental hypoxic effects. Each PHD has a different intra-cellular localization and affinity for HIF1α and HIF2α. HIF1α and HIF2α are also involved in macrophage inflammatory pathways, in which they exert both unique and opposing functions.^6^ The effect of HIF2α deficiency in atherogenesis is not studied, while the role of HIF1α is controversial.^7^^,^^8^ Thus, studying the role of their upstream regulators is important to fully elucidate hypoxic signalling in macrophages. In addition, PHDs have been shown to have HIF independent effects.^9^ Systemic protective effects of whole-body PHD1 and 2 deficiency, and pan-PHD inhibitors on mouse and human cholesterol metabolism and atherogenesis were attributed to stromal cells.^10–12^ However, the detailed mechanistic role for PHDs in plaque macrophage function remains elusive. Thus, we studied the effects of PHD protein signalling in myeloid cells on atherosclerotic plaque development and phenotype.

## 2. Methods

Fully detailed methods can be found in the Supplementary material online.

### 2.1 Animals

All mouse experiments were approved by the regulatory authority of the Maastricht University Medical Centre and performed in compliance with the Dutch governmental guidelines and Directive 2010/63/EU of the European Parliament on the protection of animals used for scientific purposes. Whole-body PHD1ko and PHD3ko mice,^13^^,^^14^ PHD2 conditional knockout mice (PHD2cko),^15^ and LysMCre transgenics^16^ were previously described and crossed to low-density lipoprotein receptor knockout (LDLrko) mice. Male LDLrko mice were used as control in all experiments involving PHD1ko and PHD3ko. LysMCre LDLrko mice (hereafter referred to as PHD2 WT) served as control in all experiments with PHD2cko mice.

### 2.2 Atherosclerosis models

For bone marrow transplantations, male LDLrko recipients were placed on antibiotic water, containing neomycin (100 mg/L; Gibco, Carlsbad, CA, USA) and polymyxin B sulfate (60.000 U/L; Gibco) for 7 weeks while being fed chow diet. After 1 week of antibiotic water, bone marrow was isolated from PHD1ko-LDLrko and PHD3ko-LDLrko mice and matched LDLrko controls (hereafter referred to as PHD1ko, PHD3ko, and WT respectively), and transplanted (1 × 10^7^ cells/mouse) into lethally irradiated LDLrko recipients (2 × 6 Gy, PHD1 *n* = 20 control vs. 20 ko, PHD3 *n* = 18 control vs. 16 ko). Mice were left to recover for 6 weeks on chow diet and subsequently placed on HCD *ad libitum* (0.25% cholesterol, SDS 824171) for 8 weeks. PHD2cko mice and respective controls (*n* = 20 per group) were fed an HCD *ad libitum* (0.25% cholesterol, SDS 824171) for 6 weeks, 12 or 20 weeks.

### 2.3. Atherosclerosis quantification and immunohistochemistry

Mice were euthanized with an intraperitoneal injection of a pentobarbital overdose (100 mg/kg) and blood was withdrawn via the right ventricle for flow cytometry and total cholesterol analysis. Mice were perfused via the left cardiac ventricle with PBS containing sodium nitroprusside (0.1 mg/mL; Sigma-Aldrich, Seelze, Germany). Aortic arch, root and organs were subsequently excised and fixed in 1% paraformaldehyde overnight and paraffin-embedded. Sections from aortic roots and brachiocephalic arteries (4 µm) were used for quantification of plaque area, necrotic core, macrophage content and cell size (MAC3), mesenchymal cell content [αSMA, platelet-derived growth factor receptor β (PDGFRβ)], collagen (Sirius red, collagen type I, SPP1), hypoxia (HIF1), proliferation (Ki67), apoptosis (TUNEL), and microvessels (CD31). More information about immunohistochemistry can be found in the Supplementary material online.

### 2.4. Flow cytometry and blood variables

Cells isolated from whole blood were analysed using flow cytometry (*n* = 10 mice per group). Blood was subjected to erythrocyte lysis. Following specific antibodies were used to detect leucocyte subsets in all tissues: leucocytes (CD45^+^, Biolegend), T cells (CD3ε^+^, NK1-1^−^; Miltenyi, eBioscience, resp.), B cells (B220^+^; BD), NK cells (NK1-1^+^), granulocytes (CD11b^high^ Ly6G^high^; BD, eBioscience, resp.), and monocytes (CD11b^high^ Ly6G^low^ Ly6C^high/intermediate/low^; Miltenyi). Data were acquired using a FACS Canto II and analysed with FACS diva software (BD).

For blood variable analysis, whole blood was diluted 1:10 in Hepes buffer, pH 7.45 (10 mM Hepes, 136 mM NaCl, 2.7 mM KCl, 2 mM MgCl_2,_ 0.1% glucose, 0.1% BSA) and subsequently measured on the XP3000 Sysmex analyzer (Sysmex, Chuo-ku Kobe, Japan).

### 2.5. Human tissue collection

Human plaque tissue collections were used: Maastricht Pathology Tissue Collection (MPTC), and Biobank of Karolinska Endarterectomies (BiKE) for analysis of protein and mRNA levels using immunohistochemistry, *in situ* hybridization, microarrays, and western blot analysis. Tissue collection was in line with the Dutch Code for Proper Secondary use of Human Tissue that is normally considered was material. This code (https://www.federa.org/codes-conduct) entails an opt-out arrangement and hence tissues were not used in case of objection. The applicability of this code for this study was approved by the Maastricht University hospital (MUMC) local Medical Ethical Committees Human studies of BiKE are approved by the Ethical Committee of Stockholm and follow the guidelines of the Declaration of Helsinki. All included patients have given their written informed consent.

### 2.6. Human plaque immunohistochemistry, *in situ* hybridization, and multispectral imaging

PHD1, 2, and 3 protein expression was assessed in human carotid autopsy samples. Slides were incubated with antibodies against PHD1 (Novus Biologicals NB100-310), PHD2 (Novus Biologicals NB100-2219) and CD68 (DAKO, M0814). Multispectral imaging was performed to analyse human PHD1 and 2 expression, and PHD-CD68 co-localization using a Nuance spectral imaging system (Perkin Elmer/Caliper Life Sciences, Hopkinton, MA, USA). As accurate, reliable PHD3 antibodies are not available at this moment in time, we used *in situ* hybridization. *PHD3* mRNA expression was determined by *in situ* hybridization in FFPE, using the following target sequence (TACATGGTGGGATCCTGCGGATAT TTCCAGAGGGGAAATCATTC ATAGCAGATGTGGAGCCCATTT TTGACAGACTCCTGTTCTTCTGGTCAGATCGTAG GAACCCACACGAAGTGCAGCCCTCTTACGCAACCAG).

### 2.7. Cell culture

Mice were euthanized by CO_2_ asphyxiation and bone marrow was isolated to generate bone marrow-derived macrophages (BMDMs). Macrophage conditioned medium was obtained by culturing differentiated BMDM for 24 h in either normal oxygen conditions or hypoxia (0.1% O_2_).

Primary vascular smooth muscle cells (SMCs) were isolated by enzymatic digestion from aortas of 5–10 C57/Bl6 mice (after removal of endothelium and adventitia) and cultured in DMEM (Gibco, 31966047) supplemented with 10% FCS and 100 U/m: Penicillin-Streptomycin. NIH/3T3 were cultured in DMEM (Gibco, 31966047) supplemented with 10% FCS and 100 u/mL Penicillin-Streptomycin. Before experiments with macrophage conditioned medium, SMCs and NIH/3T3 were starved for 24–48 h, respectively using DMEM supplemented with 0.1% FCS.

For gene intervention, cells were incubated with transfection agent Viromer BLUE (Lipocalyx, VB-01LB-01) together with siRNA for HIF1 (5′-GUCACCACAGGACAGUACA-3′), BNIP3 (5′-ACCUUCUGAUGAAGAUUUGGAUC-3′) and scramble control (5′-GCUUAACCCGUAUUGCCUA-3′) in a concentration of 25 nM for 8 h.

### 2.8. Proliferation and migration

Proliferation of SMC and 3T3 fibroblasts in response to macrophage conditioned medium (see above) was measured using CellTiter-glo luminescent cell viability assay (Promega, G7570) to determine ATP content of cells according to manufacturer’s protocol. Proliferation of PHD2 WT and cko BMDMs was also measured on an ACEA xCELLigence (Roche). Migration of primary murine SMC in response to conditioned macrophage medium of WT and PHD2cko was measured on an ACEA xCELLigence (Roche).

### 2.9. Functional assays

BMDMs were stimulated with stimuli for apoptosis [50 µM 7-ketocholesterol (Sigma, C2394) or 50 µg/mL oxLDL (isolated as described elsewhere)^17^] or lipid uptake (Topfluor, Avanti lipids 810255P-1mg). After stimulation, nuclei were stained with Hoechst (15 µg/mL). Samples were analysed using a high-throughput, fluorescent reporter system, coupled to automated microscopy (BD Pathway 855 High Content Bioimager). Data were processed with Attovision and BD Diva software. Detailed methods can be found in the Supplementary material online.

### 2.10. Efferocytosis

For efferocytosis analysis *in vitro*, Jurkat T cells were labelled with calcein-AM (1 µg/mL Invitrogen) prior to induction of apoptosis by UV irradiation (15 min 254 nm, UVS-26, 6 W bulb 0.02 J/s/cm_2_) and added 3:1 to BMDMs. Macrophages were exposed to 21% (normoxia) or 0.2% O_2_ (hypoxia) during 45 min of efferocytosis (Invivo2 1000, Ruskinn technology LTD, Pencoed, UK). After washing, macrophages were dissociated and analysed using flow cytometry for the percentage of calcein/jurkat^+^ macrophages.

### 2.11. Intracellular collagen content and collagen secretion

Intracellular collagen content of SMCs and 3T3s in response to PHD2 WT or cko conditioned medium was measured using CNA35-FITC (Kindly provided by prof. Reutelingsperger, Biochemistry department Maastricht). CNA35-FITC shown to bind to collagen type I, III and IV.^18^ Samples were analysed using the BD Pathway 855 High Content Bioimager. Data were processed with Attovision and BD Diva software.

SMCs and 3T3s were treated with PHD2 WT or cko conditioned medium for 72 h. Culture medium of SMCs and 3T3s was collected and analysed using Sircol soluble collagen assay as described by the manufacturer (Biocolor, S1000). In comparable subsequent experiments, transcription growth factor (TGF)-β1 was added to the conditioned medium or proteins were heat-inactivated (30 min, 85°C) prior to addition to 3T3 fibroblasts.

### 2.12. Matrix metalloprotease activity assay

The functional activity of matrix metalloproteinase (MMP) was determined using OmniMMP™ fluorogenic substrate (Enzo Life Science, BML-P126-0001). Fluorescence was detected at an interval of two minutes on a Spectromax (Ex 328 nm, Em 393 nm, Molecular Devices SPECTRAmax M2).

### 2.13. Western blot analysis

Whole-cell BMDM protein lysate was isolated using RIPA buffer supplemented with protease inhibitors (Roche, 11873580001) and protein concentration determined by BCA assay.

Pre-cast gels (ExpressPlus PAGE gel 8–16%, genscript, M81612) were used to and proteins were transferred to a nitrocellulose membrane. Primary antibodies directed against HIF1α (Novus Biologicals, NB100-499), HIF2α (Novus Biologicals, NB100-122), and β-Actin (Abcam, ab8227), were followed by appropriate HRP-labelled secondary antibody incubation. Signal was developed using SuperSignal West Femto Maximum Sensitivity Substrate (Thermo Fisher scientific, 34095) and visualized using a digital scanner. Band density was quantified with ImageJ, and normalized for total proteins by β-actin as loading control.

### 2.14. Real-time quantitative PCR

*In vitro* experiments for gene expression analysis were performed in quadruplicate and repeated twice. Total RNA was isolated by Qiazol (Qiagen, 79306) and transcribed using iSCRIPT (Biorad, 1708891). qPCR analyses were performed with 10 ng cDNA using SYBR green (Biorad) and gene-specific primer sets (Eurogentec, Liege, Belgium, Supplementary material online, *Table S1*). Two housekeeping genes (cyclophilin, 18S rRNA) were used to normalize differences in mRNA levels between samples.

### 2.15. RNA sequencing and bioinformatic analysis of cultured cells

For RNA sequencing cells *in vitro*, RNA was isolated from triplicates of WT and PHD2cko BMDMs 24 h after seeding, and from triplicate fibroblasts after 72 h exposure to WT or PHD2cko conditioned medium. Bioanalyzer confirmed intact RNA (RNA Integrity number 10) for sequencing of 10 µg RNA, on the NextSeq 500 system using v 2.5 chemistry, at ∼15 M single reads per sample by the c (Core Facility Genomics of the Medical Faculty Münster). The gene-level expression of a total of 32 544 genes were quantified, 14 285 genes were retrieved for downstream analysis, 18 259 genes were discarded as lowly expressed genes. Data analysis was performed in R (stats package, R.3.6.1 version). Full information on bioinformatic analysis can be found in the Supplementary material online.

### 2.16. Single-cell RNA sequencing of murine plaques

Single-cell sequencing was performed on PHD2 WT and PHD2cko plaques (*n* = 11 and 9, respectively). Aortic arches of either genotype were pooled, enzymatically digested and subjected to red blood cell lysis. All single, DAPI-negative living, cells were sorted on FACS Aria III for SCS. Cells were loaded on a chromium single-cell controller using V2 reagent kit (10× Genomics) to create cDNA sequencing libraries per manufacturers protocol. The Chromium Single-Cell 3′ Library Kit was then used to generate indexed sequencing libraries. Sequencing was performed on a Novaseq 6000 system (Illumina) (Supplementary material online, *Table S2*).

### 2.17. Bioinformatics analysis of single-cell RNA sequencing data

Raw sequencing data were processed using CellRanger and analysed using R (R.3.6.1), Seurat R package (v.3.1.0), and Bioconductor R packages. Full details of analysis can be found in the Supplementary material online.

### 2.18. Statistical analysis

All data are presented as mean ± standard error of the mean, or effect size, with *P-value <0.5, **P-value < 0.01, ***P-value < 0.001. All variables were analysed using independent sample tests and were tested for normal distribution using Shapiro–Wilk normality test. Variables with two groups were compared with Student’s *t*-test or Mann–Whitney rank-sum test. In case of more than two groups, variables were analysed using one-way analysis of variance followed by Bonferroni’s Multiple Comparison Test or Kruskal–Wallis rank-sum test, followed by Dunn’s *post hoc* testing. Correlation analysis was performed using Spearman bivariate correlation analysis (IBM SPSS statistics 22).

## 3. Results

### 3.1 PHD1, 2, and 3 in human plaque correlates with plaque inflammation

**Figure 1:**
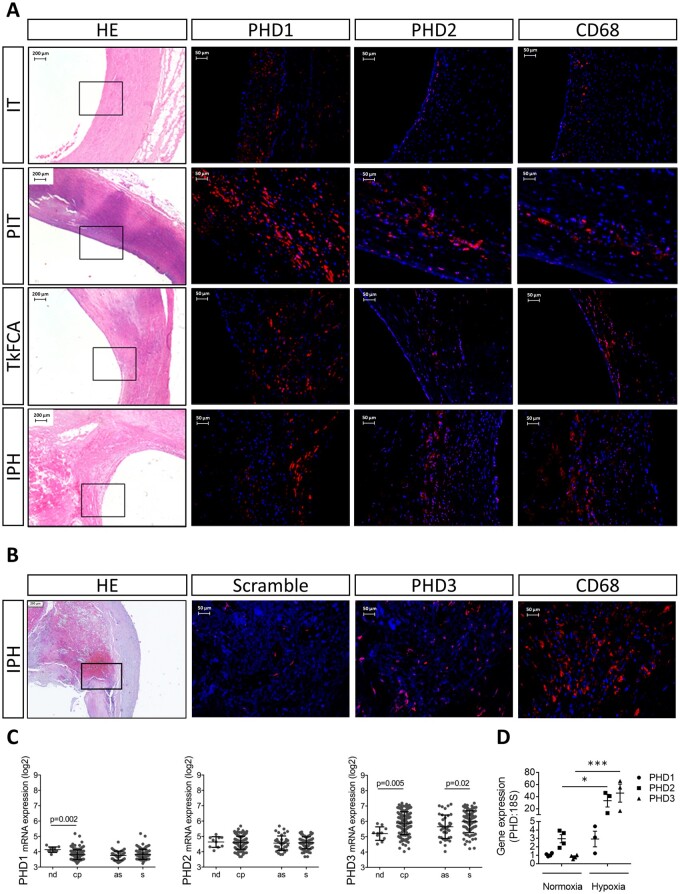
PHDs correlated with in human plaque inflammation. (*A*) Representative pictures of haematoxylin/eosin (HE), PHD1, PHD2, and CD68 in adjacent human carotid plaque sections from different stages [intimal thickening (IT), pathological intimal thickening (PIT), thick fibrous cap atheroma (TkFCA), and intraplaque haemorrhage (IPH)], (*B*) Representative pictures of HE, Scramble, PHD3 *in situ* hybridization, and CD68 immunoreactivity in IPH human carotid plaque. (*C*) PHD mRNA expression in microarrays of non-diseased arteries (nd, *n* = 10) and carotid plaques (cp, *n* = 127) from the BiKE cohort. Carotid plaques were further stratified as asymptomatic (as, *n* = 40) or symptomatic patients (s, *n* = 87). (*D*) PHD isoforms mRNA expression in bone marrow-derived macrophages (BMDMs) measured by quantitative PCR (*n* = 3–4 replicates). Expression relative to 18S. Statistical analyses were performed using two-way ANOVA, with Bonferroni *post hoc* test (*C* and *D*). All results show mean ± SEM. *P < 0.05, ***P < 0.001.

Protein (PHD1, 2) and mRNA (PHD1, 2, 3) expression was studied in serial sections of atherosclerotic tissue of human carotid arteries to establish cell-type and plaque stage expression patterns of each isoform. All PHDs were expressed throughout different stages of atherosclerosis (*Figure 1A*–*C*). PHD1 was expressed in both CD68 positive and negative regions (*Figure 1A*), while PHD2 and 3-localized predominantly with regions that were CD68-positive in serial sections (*Figure 1A and B*). Consistent with the importance of inflammation and macrophage function for plaque stability, this suggested that PHDs could impact plaque stability. We therefore analysed PHD expression in the large human cohort BiKE, with both healthy and atherosclerotic carotid arteries. Similar to protein expression, mRNA expression patterns of the three PHD isoforms differed between disease stages. PHD1 and PHD3 mRNA levels were increased between non-diseased arteries and atherosclerotic plaques. PHD1 and PHD2 mRNA was similar between plaques from clinically symptomatic and asymptomatic patients, while PHD3 mRNA was increased in plaques from symptomatic patients (*Figure 1C*). The link with inflammation and hypoxic signalling was confirmed by the significant correlations between *PHD* mRNA and *CD68,* and *HIF1α* and *HIF2α* in samples of BiKE cohort subjects (Supplementary material online, *Table S2*). Thus, expression of PHD proteins in CD68-rich regions in human plaque tissue suggests that PHDs may modulate atherosclerosis via the macrophage compartment.

### 3.2 Myeloid PHD1, 2, and 3 deficiency leads to different plaque phenotypes

**Figure 2:**
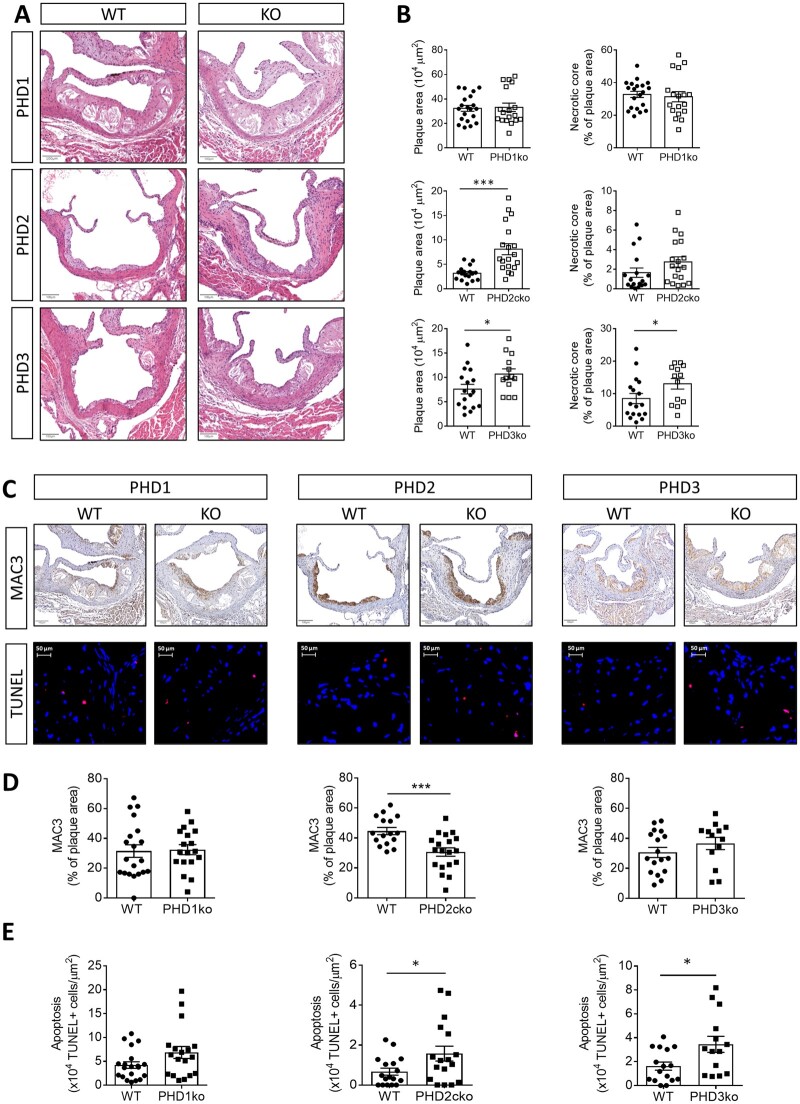
Myeloid PHD2 and PHD3 deficiency aggravated plaque apoptosis. (*A*) Representative HE pictures of aortic root (AR) lesions in PHD knock-outs (KO) and corresponding controls (WT) after 6–8 weeks high-cholesterol diet. (*B*) Quantification of plaque and necrotic core size. (*C*) Representative pictures of MAC3 (brown) and TUNEL (red) staining in ARs of all PHD KO models and corresponding quantification (*D, E*, respectively). Statistical analyses were performed using a Student’s *t*-test (*B, D, E*). All results show mean ± SEM of 13–20 mice per group. *P < 0.05, ***P < 0.001. Scale bars 100 µm.

The different expression patterns of PHDs in human plaques, and murine BMDMs (*Figure 1D*) warranted investigation of each isoform in atherogenesis. As only PHD2 is embryonically lethal, PHD2 LysMCre conditional knockout (ko) mice on a low-density lipoprotein receptor (LDLR) ko background (PHD2cko) and LysMCre-LDLRko (WT) were fed a high-cholesterol diet to study atherogenesis. Transplantations of LDLRko (WT), PHD1ko-LDLR-ko, and PHD3ko-LDLR-ko bone marrow into LDLRko recipient mice ensured knockout in myeloid cells (Supplementary material online, *Figure S1A*–*C*). Both PHD2cko and PHD3ko led to enhanced plaque size in the aortic root, while PHD3ko additionally showed increased necrotic content (*Figure 2A and B*). Interestingly, plaques in PHD1ko mice were similar to control, despite a small decrease in plasma cholesterol (Supplementary material online, *Figure S1D*). This is surprising, given that whole body PHD1ko and PHD2 hypomorphous mice showed an atheroprotective metabolic phenotype, and smaller plaques compared to control mice.^10^^,^^11^ However, PHD1 expression was less pronounced in murine and human macrophages compared to stromal cells, and to macrophage PHD2 and PHD3 (*Figure 1*). This may explain a preferential stromal, not myeloid, role for PHD1, consistent with its role in extrahepatic cholesterol metabolism.^10^ Importantly, no major systemic changes in body weight or blood leucocytes occurred in PHD2 and PHD3 deficient mice (Supplementary material online, *Figures S1E* and *F, S2A*–*C*). We observed no difference in the numbers of circulating neutrophils in all knock-out models or neutrophil presence in PHD2cko and PHD3ko plaques (Supplementary material online, *Figures S2A.6, B.6, C.6, D* and *E*).

### 3.3 Myeloid PHD2 and PHD3 deficiency increased macrophage apoptosis

**Figure 3:**
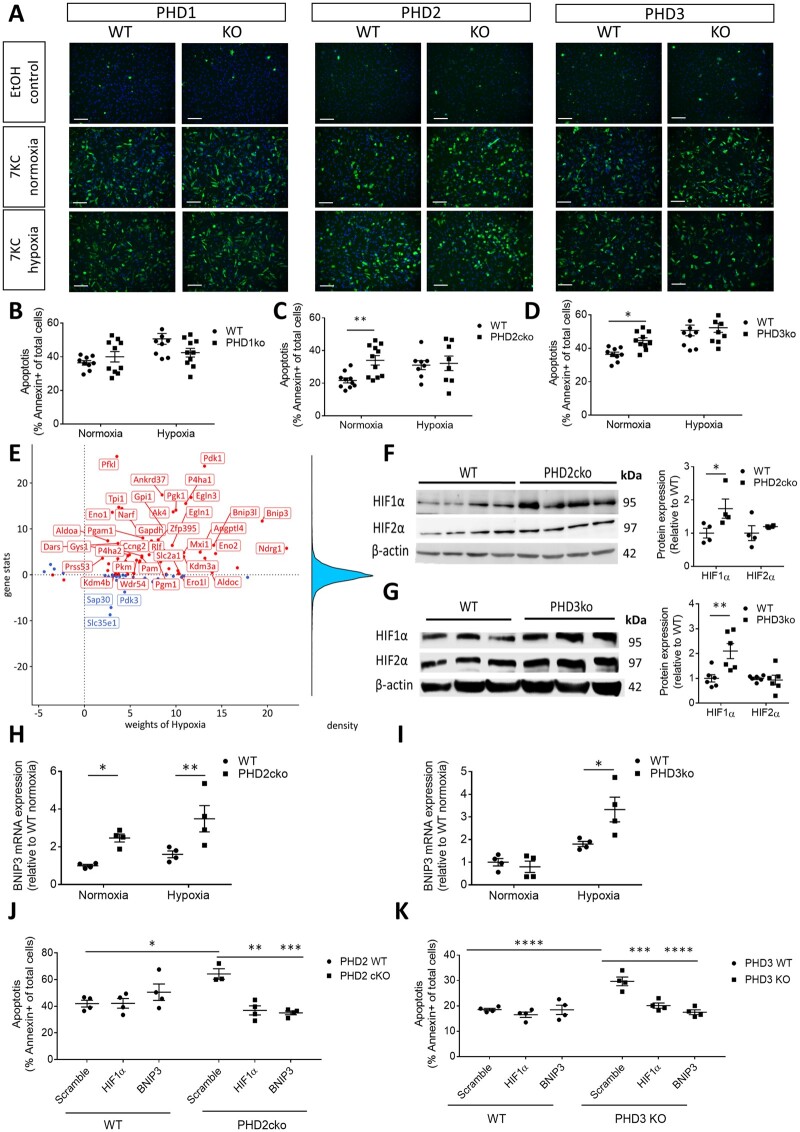
PHD2cko and PHD3ko enhanced BMDM apoptosis via a HIF1/BNIP3 axis. (*A*) Representative pictures Annexin-A5 (green) stained BMDMs after ethanol (EtOH) or 7-ketocholesterol (7KC) stimulation in normoxia or hypoxia. Nuclei in blue. Bar is 20 µm. Graphs show 4–11 experiments, which consist of 3–6 biological replicates per experiment (*B*–*D*). Corresponding quantification of percentage apoptotic cells per isoform. (*E*) PROGENy footprint of hypoxia response in PHD2cko macrophages. Top 10% most dysregulated genes are shown. Red/blue: positive/negative contribution to activity. (*F*) Western blot and quantification of HIF1α and HIF2α expression in PHD2cko, (*G*) PHD3ko and respective control BMDM, normalized for β-actin. Full blots can be found in Supplementary material online, *Figure S6*. (*H*) BNIP3 mRNA expression in PHD2cko and (*I*) PHD3ko BMDM under normoxic and hypoxic conditions. (*J*) Apoptosis quantification of PHD2cko (*K*) and PHD3ko BMDMs treated with either scramble, HIF1α or BNIP3 siRNA and 7KC. Experiments show four to six biological replicates. Statistical analyses were performed using a two-way ANOVA with Bonferroni *post hoc* test (*B*–*D, F*–*K*). All results show mean ± SEM. *P < 0.05, **P < 0.01, ***P < 0.001.

Myeloid-specific PHD loss led to reduced macrophage content in PHD2cko plaques and larger necrotic cores in PHD3ko plaques, but not PHD1ko plaques (*Figure 2C and D*). As reduced plaque macrophage numbers and larger necrotic core size can both result from increased macrophage apoptosis. We therefore assess apoptosis by TUNEL staining and revealed enhanced macrophage apoptosis in PHD2cko and PHD3ko plaques (*Figure 2C*–*E*). Furthermore, PHD2cko and PHD3ko BMDMs, but not PHD1ko BMDMs, showed enhanced 7-ketocholesterol induced annexin V-based apoptosis *in vitro* (*Figure 3A*–*D*). PHD2cko BMDM responded similarly to oxLDL (Supplementary material online, *Figure S3A* and *B*). Uptake of apoptotic cells *in vitro* was not different between PHD2cko, PHD3ko, and control cells (Supplementary material online, *Figure S3C* and *D*), suggesting apoptosis alone is underlying enhanced density of apoptotic cells rather than efferocytosis.

### 3.4 Apoptosis *in vitro* was enhanced via HIF1α and BNIP3

Transcriptome-wide mRNA profiling of PHD2cko BMDMs was performed to study underlying mechanisms (*Figure 3E* and Supplementary material online, *Figure S4*). Pathway analysis using PROGENy,^19^ and transcription factor analysis using DoRothEA^20^ in PHD2cko BMDMs supported hypoxia response activation and HIF1α transcription factor activity. Hallmarks of cell division, and glycolysis were also up-regulated, while inflammatory signalling was diminished (*Figure 3E*, Supplementary material online, *Figure S4B*–*D, Tables S3* and *S4*). In contrast, *in vitro* PHD2cKo showed up-regulated expression of both pro- and anti-inflammatory genes (Supplementary material online, *Figure S4E*). Interestingly, expression of the pro-apoptotic HIF target gene BCL2/adenovirus E1B 19 kDa protein-interacting protein 3 (BNIP3) was up-regulated (1.8-fold-change, FDR < 0.05; *Figure 3E*, Supplementary material online, *Figure S4C*). Culturing control BMDMs under hypoxic conditions, mimicking PHD deficiency, led to increased apoptosis to comparable levels as in PHD-deficient BMDMs (*Figure 3A*–*D*). PHD2cko and PHD3ko BMDMs did not show further augmentation of apoptosis upon hypoxic conditions (*Figure 3A*–*D*). Increased HIF1α, but not HIF2α protein levels in PHD2cko and PHD3ko BMDMs and plaques (*Figures 3F and G*, Supplementary material online, *Figure S5A* and *S6*) was accompanied by enhanced BNIP3 mRNA expression in normoxic and hypoxic PHD2cko and PHD3ko BMDMs (*Figure 3H*–*J*). The causal involvement of HIF1α, and BNIP3 in macrophage apoptosis was confirmed by silencing HIF1α, or BNIP3 in PHD2cko and PHD3ko BMDMs, significantly decreasing apoptosis compared to scramble siRNA-treated control cells (*Figure 3J and K*, Supplementary material online, *Figure S5B* and *C*).

### 3.5 Single-cell sequencing confirmed up-regulated HIF1α/BNIP3 in PHD2cko plaque macrophages *in vivo*

**Figure 4:**
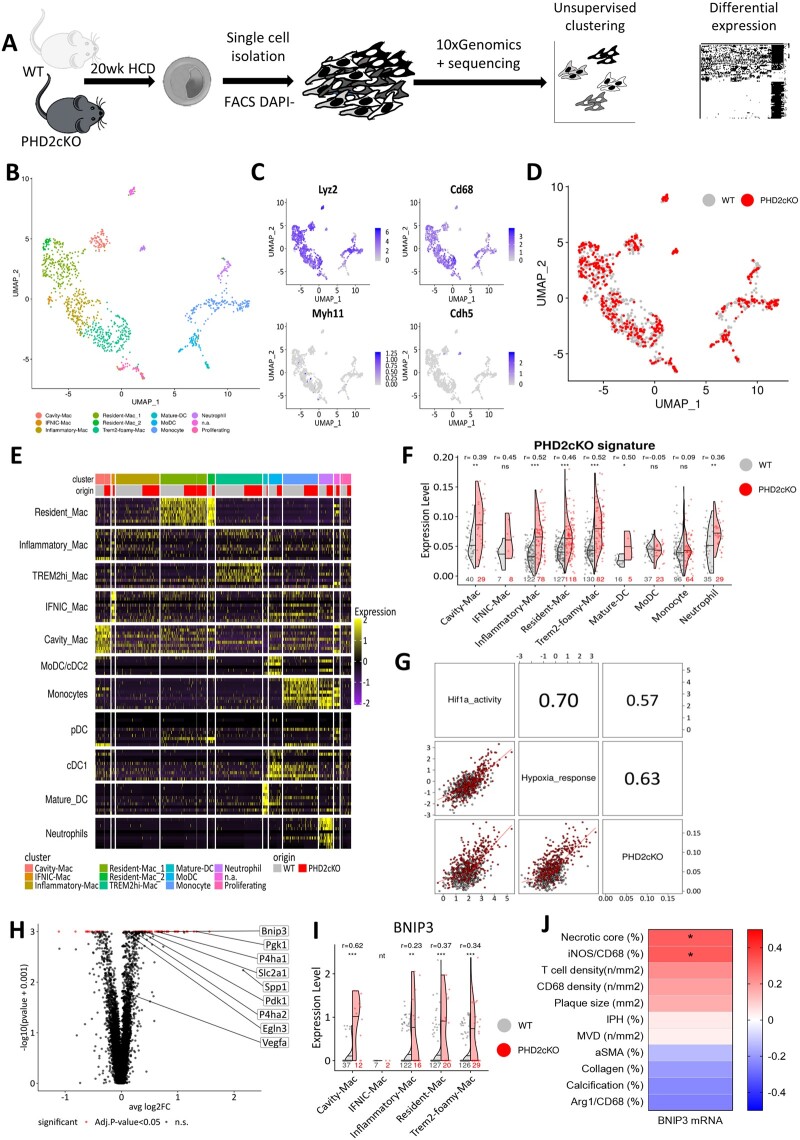
Single-cell sequencing of PHD2cko plaques supported hypoxia signalling and BNIP3 up-regulation *in vivo*. (*A*) Schematic overview single-cell sequencing setup, 11 PHD2 WT and 9 PHD2cko mice were pooled for single-cell sequencing. (*B*) Uniform Manifold Approximation and Project (UMAP) plot of the myeloid leucocytes (*n* = 1119). Unsupervised clustering resulted in 12 clusters, whose identities were assigned based on cell-specific markers as shown in (*E*). Each dot represents a single cell, grouped together based on similarities in transcriptome. (*C*) Same UMAP plot as in (*B*) showing the absolute expression of canonical markers for myeloid (Lyz2 and CD68), and non-myeloid lineages (Myh11 and Cdh5). (*D*) Same UMAP plot as in (*B*) colour by biological condition of origin. (*E*) Heatmap of the top 10 markers from Zernecke *et al*. of each myeloid leucocyte class across cells. Columns are grouped by the resulting 12 clusters from the unsupervised clustering. Columns are single cells. Rows are marker genes. Gene-level expression was scaled across cells. (*F*) Violin plots with group 50th percentile (horizontal line) split by condition showing differences in the expression of the BMDM PHD2cKO expression signature among myeloid leucocytes. Each dot represents a cell. ***P < 0.001, **P < 0.01, *P < 0.05 adjusted *P*-values (FDR method) and *r* effect sizes from Wilcoxon test. Sample sizes are indicated at the bottom of each violin group. (*G*) Correlation matrix showing pair-wise Pearson correlation coefficients between PHD2cko signature and hypoxia and HIF1α activity scores at a single-cell basis. WT and PHD2cko cells in grey and red respectively. (*H*) Volcano plot depicting differentially expressed genes between PHD2cko and WT plaque macrophages with high (>3Q, *n* = 79 cells) and low (<Q3, *n* = 419 cells) expression of the BMDM PHD2cKO expression signature, respectively. DEGs with adj. *P*-value <0.05 (Bonferroni method) in red. (*I*) Violin plots split with group 50th percentile (horizontal line) by condition showing differences in the Bnip3 expression between PHD2cko and WT plaque macrophages with high and low expression of the BMDM PHD2cKO expression signature, respectively. Each dot represents a cell. ***P < 0.001, **P < 0.01, *P < 0.05 adjusted *P*-values (FDR method) and *r* effect sizes from Wilcoxon test. Sample sizes are indicated at the bottom of each violin group. IFNIC-Mac was not tested (nt) due to low sample size (*n* < 5). (*J*) Heatmap showing FDR adjusted Pearson correlation of BNIP3 mRNA in human carotid plaque segments (*n* = 22 patients) with plaque traits in adjacent sections. *FDR adjusted *P*-value <0.05.

As transcriptional effects of cell-type conditional silencing with partial knockdown can be diluted by other cell types in ‘bulk’ RNA sequencing, single-cell RNA sequencing of plaque macrophages was essential to distill the transcriptome of plaque macrophages with PHD2cko (*Figure 4A*). The single-cell map showed the landscape of myeloid leucocytes described previously in atherosclerotic plaques (*Figure 4B*).^21^ These myeloid cells were Lyz2-positive, but negative for SMC and endothelial cell markers (*Figure 4C*). Inflammatory, resident-like, ‘triggering receptor expressed on myeloid cells-2’ (TREM2hi), interferon-inducible cell (IFNIC) and cavity macrophages, a recently-discovered subset of macrophages resembling those from the peritoneal cavity,^22^ were clearly identified and represented similarly in both genotypes (*Figure 4D and E*, Supplementary material online, *Figure S7A*). PHD2cko plaque macrophages were identified based on the PHD2cko gene signature, derived from the *in vitro* transcriptome of PHD2cko BMDMs. The top 50 signature genes, including amongst others Pyruvate Dehydrogenase Kinase 1 (Pdk1) and Prolyl 4-Hydroxylase Subunit Alpha 1 (P4ha1), can be found in Supplementary material online, *Table S5*. Myeloid cells derived from PHD2cko plaques presented with a greater PHD2cko gene signature expression as compared to their matched WT cells (*Figure 4F*). Next, PHD2cko cells with signature expression above the 3rd quartile (Q3) from PHD2cko plaque macrophages (*Figure 4F*) were compared to WT plaque macrophages below Q3 for differential gene and pathway analysis. This confirmed hypoxia/HIF signalling upon PHD2cKO (*Figure 4G*), using PROGENy and DoRothEA methods on single cells,^23^ and BNIP3 overexpression in PHD2cko plaque macrophages *in vivo* (*Figure 4H and I*). BNIP3 expression was increased across all macrophage subsets. In contrast, BNIP3 was not differentially expressed in PHD2cKO plaque neutrophils with high expression of the PHD2cKO signature (Supplementary material online, *Figure S7B*). Furthermore, we were able to show the correlation of BNIP3 mRNA, derived from microarrays of human carotid plaques, with necrotic core size (*Figure 4J*). Taken together, the shows that the PHD2/3-HIF1α-BNIP3 axis likely explains enhanced plaque apoptosis and the reduction in plaque macrophages observed in PHD2cko plaques.

### 3.6 PHD2cko, but not PHD1/3ko, promoted a pro-fibrotic plaque phenotype

**Figure 5:**
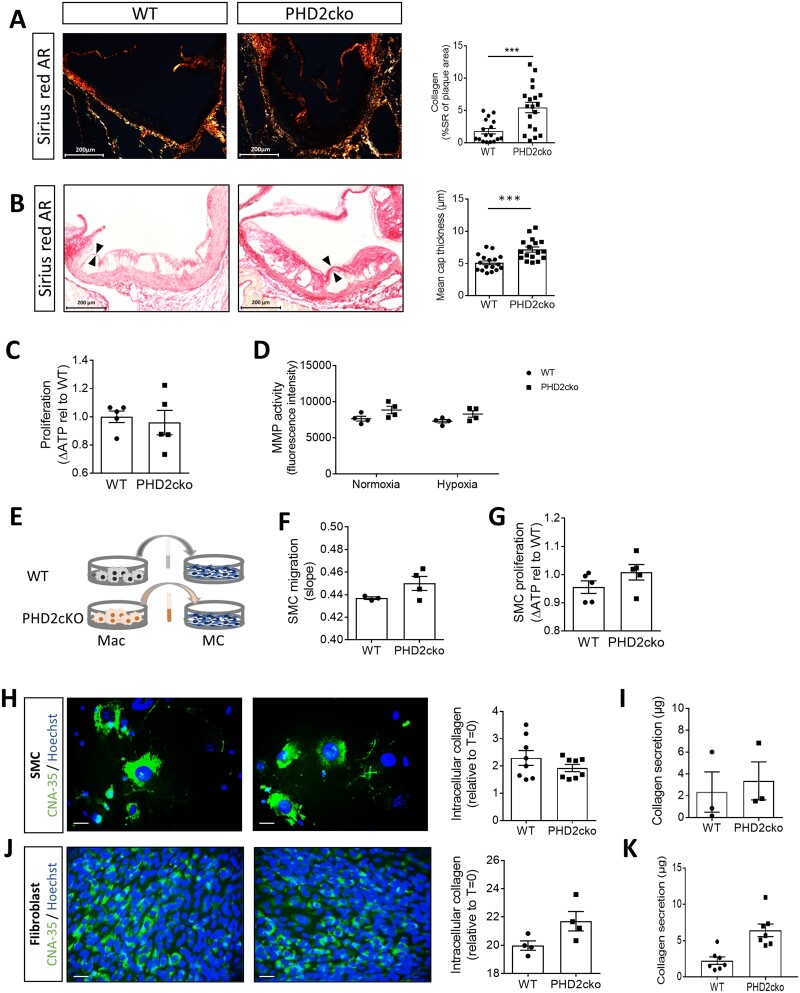
Myeloid PHD2 deficiency triggered plaque fibrosis by enhancing fibroblast collagen secretion via PHD2cko BMDM paracrine signalling. (*A*) Representative pictures of Sirius red collagen content in PHD2cko and WT mice after 6 week HCD. (*B*) Fibrous cap thickness (arrows) in PHD2cko and WT mice after 12 weeks HCD. (*C*) BMDM proliferation (ATP accumulation over 72 h) and (*D*) MMP activity of PHD2cko and WT BMDMs in normoxia and hypoxia. (*E*) Schematic overview of conditioned medium transfer from BDMDs to mesenchymal cells (MCs). (*F*) SMC migration after conditioned medium transfer. (*G*) SMC proliferation (ATP accumulation) h after conditioned medium transfer. (*H*) Representative pictures and quantification of internal collagen (CNA-35, green) in SMCs conditioned medium transfer. Bar is 20 µm. (*I*) SMC collagen secretion after conditioned medium transfer. (*J*) Representative pictures and quantification of internal collagen in 3T3 fibroblasts measured by CNA-35 (green) after 72 h conditioned medium transfer. Bar is 20 µm. (*K*) 3T3 fibroblast collagen secretion after conditioned medium transfer. *In vitro* experiments were done with three to five technical replicates, and three to six biological replicates. *In vivo* studies include 17–20 mice per group. Statistical analyses were performed using a Student’s *t*-test (*A*–*C, F*–*K*) or two-way ANOVA, with Bonferroni *post hoc* test (*D*). All results show mean ± SEM. *P < 0.05, **P < 0.01, ***P < 0.001.

Despite increased apoptosis, larger plaques were observed in PHD2cko and PHD3ko mice. In PHD2Ko mice, this was mostly attributed to a ∼3-fold higher collagen content (*Figure 5A*), while neither PHD1ko nor PHD3ko mice showed enhanced collagen content (Supplementary material online, *Figure S8*). Plaque size and collagen accumulation of PHD2cko mice were both significantly enhanced in advanced plaques in the aortic root, and the brachiocephalic artery in a further cohort of mice fed a high-cholesterol diet for 12 weeks (Supplementary material online, *Figure S9A*–*D*). In addition to overall collagen accumulation, cap-thickness was increased (*Figure 5B*). Of note, micro-vessel density was not altered in the adventitial tissue of the aortic root and brachiocephalic artery of PHD2cko animals, although PHD2 was reported to be the main isoform driving angiogenesis.^24^ Additionally, no plaque micro-vessels were observed in WT or PHD2cko mice, as expected in mice (Supplementary material online, *Figure S9E* and *F*).

### 3.7 Paracrine signalling by PHD2cko macrophages enhanced fibroblast collagen secretion

Collagen secretion by BMDMs themselves was undetectable (Supplementary material online, *Figure S9G*). Additionally, matrix metalloproteinase activity, and proliferation of PHD2cko BMDMs were unchanged, as measured by two independent assays (*Figure 5C and D*, data not shown). This hinted towards paracrine effects of myeloid PHD2cko on density or function of collagen-producing cells. Although the current dogma suggests that smooth muscle cells (SMC) are the source of plaque collagen, *in vitro* and *in vivo* SMC migration, proliferation, and collagen production remained similar (*Figure 5E*–*I*, Supplementary material online, *Figure S10A*). Although the area of αSMA-positive mesenchymal cells, i.e. SMCs and fibroblast-like cells, in the plaque was increased in early plaques of PHD2cko mice, this difference was not maintained at all timepoints and vascular beds (Supplementary material online, *Figure S10B* and *C*). Together these data suggests that SMC function and density were not altered by paracrine signalling of PHD2cko macrophages.

Therefore, we studied fibroblasts as source of plaque collagen accumulation, since three recent reports indicated fibroblasts involvement in atherosclerosis.^25–27^ Indeed, medium conditioned for 24 h by PHD2cko macrophages (PHD2cko-condtioned medium) induced an almost three-fold enhanced collagen secretion by 3T3 fibroblasts *in vitro*, compared to WT-conditioned medium, while intracellular collagen content remained unchanged (*Figure 5J and K*). Fibroblast density seemed unaffected, as mesenchymal marker PDGFR β content of WT and PHD2cko plaques was similar (Supplementary material online, *Figure S10D*), as was fibroblast proliferation and myogenic transition in conditioned medium *in vitro* (data not shown; Supplementary material online, *Figure S10E*). Thus, the increased collagen content in response to PHD2cko macrophages appears to relate to perturbations in fibroblast function, rather than proliferation or myogenic transitioning.

**Figure 6:**
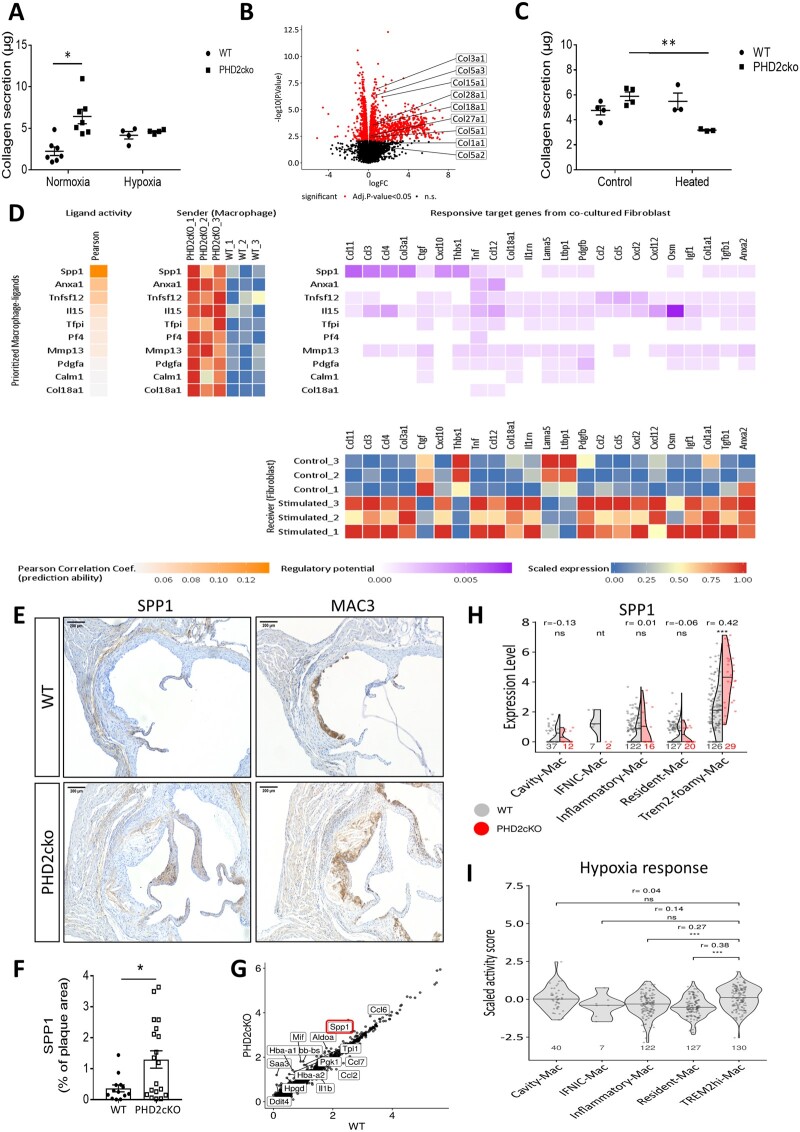
PHD2cko macrophage-fibroblast communication via SPP1 enhanced collagen accumulation. (*A*) Collagen secretion by 3T3 fibroblasts after incubation with conditioned medium of normoxic or hypoxic WT and PHD2cko BMDMs. (*B*) Volcano plot highlighting collagen genes up-regulated in 3T3 fibroblasts exposed to PH2cko-conditioned medium vs. WT. (*C*) Collagen secretion of 3T3 fibroblasts upon heat-inactivated conditioned medium exposure from WT- and PHD2cko BMDMs compared to control condition. (*D*) Nichenet sender-receiver analysis of macrophage-fibroblast communication. BMDM-derived genes (yellow) are ranked by predictive probability of 3T3 fibroblast transcriptomic changes. Fibroblast genes (purple) are ranked by correlation to the predicted BMDM trigger gene. Heatmaps of gene expression in WT and PHD2cko BMDM, and WT and PHD2cko exposed fibroblasts, confirmed differential expression of predicted targets. (*E*) Representative images of SPP1 and MAC3 immunoreactivity in WT and PHD2cko ARs after 6 weeks of HCD. (*F*) Quantification of SPP1 immunoreactivity in WT and PHD2cko ARs. (*G*) Scatterplot of log fold change expression between PHD2cKO and Wt macrophages, highlighting SPP1 as one of the most up-regulated genes. (*H*) Violin plots with group 50th percentile (horizontal line) split by genotype showing differences in the Spp1 expression between PHD2cko (orange) and WT (grey) plaque macrophages with high (>Q3) and low expression (<Q3) of the BMDM-derived PHD2cKO signature, respectively. Each dot represents a cell. ***P < 0.001, **P < 0.01, *P < 0.05 adjusted *P*-values (FDR method) and r effect sizes from Wilcoxon test. Sample sizes are indicated at the bottom of each violin group. IFNIC-Mac was not tested (nt) due to low sample size (*n* < 5). (*I*) PROGENy hypoxia response activity in macrophage subsets. *In vitro* experiments done with three to five technical replicates, and repeated three to six times. *In vivo* studies include 13–19 mice per group. Statistical analyses were performed using a two-way ANOVA, with Bonferroni *post hoc* test (*A, C*) or a Student’s *t*-test (*H*). All results show mean ± SEM. *P < 0.05, ***P < 0.001.

Mechanistically, the collagen accumulation was hypoxia-dependent, as hypoxia increased collagen production by fibroblasts treated with WT-conditioned medium to similar levels as exposure to PHD2cko-conditioned medium, without further enhancing collagen production in PHD2cko-conditioned medium treated fibroblasts (*Figure 6A*). To further decipher the molecular mechanism, the transcriptome of fibroblasts incubated *in vitro* with WT or PHD2cko medium was interrogated (Supplementary material online, *Figure S11A* and *B*). In line with *in vivo* and *in vitro* collagen accumulation, gene-set enrichment analysis showed up-regulation of collagen genes (*Figure 6B*, Supplementary material online, *Table S6*), while pathway analysis, using PROGENy,^19^ showed that the well-known pro-fibrotic transcription growth factor beta (TGFβ) signalling was down-regulated (Supplementary material online, *Table S7*). In line, addition of TGFβ to WT-conditioned medium did not mimic the enhanced collagen secretion by PHD2cko-conditioned medium, consistent with similar TGFβ gene expression by WT and PHD2cko macrophages (Supplementary material online, *Figure S11C* and *D*).

Heat-inactivation of proteins in the conditioned medium ablated collagen accumulation in response to PHD2cko-conditioned medium firmly suggesting a protein-driven paracrine effect (*Figure 6C*). Thus, transcriptomics were analysed to delineate macrophage-fibroblast communication *in vitro* using Nichenet.^28^ Nichenet identified ligands expressed by macrophages, best predicting the observed changes in signalling response genes expressed by fibroblasts (*Figure 6D*). The most likely candidate was secreted phosphoprotein 1 (SPP1), also known as osteopontin, which has a well-known correlation with cardiovascular events and fibrosis.^29^^,^^30^ Importantly, PHD2cko plaques showed increased SPP1 protein expression *in vivo*, which was overlapping but not restricted to MAC3 positive areas (*Figure 6E and F*). Further, all plaque macrophages—without subset divisions- showed a trend to increased SPP1 mRNA expression in single-cell analysis, while SPP1 mRNA was significantly up-regulated only in TREM2hi PHD2cko macrophages (*Figure 6G and H*). Interestingly, TREM2hi foamy macrophages showed increased expression of the hypoxia signalling pathway in WT macrophages (*Figure 6I*). Nevertheless, the total TREM2hi gene signature was not enhanced by PHD2cko, only SPP1 expression was increased (Supplementary material online, *Figure S11E* and *F*). In line, PHD2cko macrophages are smaller *in vivo*, and show reduced lipid uptake *in vitro* (Supplementary material online, *Figure S11G* and *H*) excluding an effect on foam cell properties, normally attributed to TREM2hi macrophages. The TREM2 hi macrophages exhibited a selective change in SPP1 expression, which could be important in mediating the pro-fibrotic plaque phenotype. Of note, SPP1 was not detected as differentially expressed genes in plaque neutrophils upon PHD2cKO perturbation (Supplementary material online, *Figure S6B*). These results underline that fibroblasts are likely the major target for paracrine pro-fibrotic signalling mostly by PHD2cko macrophages, possibly mediated by SPP1 derived from TREM2hi macrophages.

## 4. Discussion

In this study, the effect of PHD-dependent pathways on myeloid hypoxic signalling in the atherosclerotic plaque was assessed. Our study demonstrates that myeloid deficiency of PHD2 and PHD3, but not PHD1, increases plaque size and alters plaque phenotype via intracellular and paracrine signalling mechanisms. Intracellular signalling in PHD2cko and PHD3ko increased macrophage apoptosis in a HIF1α/BNIP3 axis-dependent manner, an effect observed across macrophage subsets. Our studies unveiled paracrine signalling of PHD2cko-TREM2hi macrophages to fibroblasts, promoting the latter towards a pro-fibrotic phenotype.

The pro-apoptotic effect of PHD2cko in macrophages seemed dependent on a lipidaemic background, as PHD2cko BMDMs originating from mice on normolipidaemic background did not show changes in apoptosis upon incubation with non-lipid apoptotic stimuli.^31^ Nevertheless, a microarray of these PHD2cko BMDM also showed up-regulated BNIP3 expression,^31^ consistent with our findings. Although the relationship between HIF signalling and BNIP3 activation is known from carcinogenesis, the role of BNIP3 in context of atherosclerosis has not been described before. Moreover, we show that expression of BNIP3 mRNA is associated with human plaque necrosis and pro-inflammatory macrophages. Future studies are warranted to determine if BNIP3 deficiency blocks atherogenesis, and if inhibition is a viable new therapeutic option.

Despite increased apoptosis, plaques in PHD3ko and PHD2cko mice were not smaller, as one might expect in early lesions and intact clearance of apoptotic cells.^32^ Additionally, in PHD3ko mice macrophage surface area was even larger (data not shown), while content (% surface area) was unchanged. This may be explained by increased macrophage adhesion and/or migration, as we observed similar proliferation, lipid uptake, and -efflux *in vitro* (data not shown), and no increased size of other plaque components. Indeed, HIF1 has been shown to induce monocyte adhesion upon acute hypoxia.^33^ Macrophage migration upon PHD3 ko was shown unaffected by Beneke *et al*.^34^ Nevertheless, enhanced necrotic core content was in line with stimulation of apoptosis.

In contrast, myeloid PHD2 deficiency enhanced plaque fibrosis may readily explain increased plaque size. Unlike a recent study supporting transition of macrophages into a collagen-producing, fibroblast-like subtype,^37^ we uncovered a paracrine, pro-fibrotic effect on fibroblasts. The prevailing dogma is that SMCs are the dominant source of matrix production in atherosclerosis, yet we did not observe any changes in SMC function upon PHD2cko conditioned medium transfer. Far less is known about the function of fibroblasts in atherosclerosis, however, two recent studies have pointed at the contribution of fibroblast-like cells to matrix production in atherosclerosis,^25^^,^^26^ and a recent single-cell sequencing study described two fibroblast types and an intermediate modulated SMC in human and murine atherosclerosis.^27^

Here, we also show evidence for paracrine signalling of macrophages to fibroblasts likely mediated by SPP1. SPP1 is a matricellular protein expressed by most vascular cells, and its function depends on disease state and duration.^36^ This likely explains divergent data of reduced collagen in smaller plaques of atherogenic, normoglycaemic SPP1ko mice, contrasting with enhanced vascular calcification in advanced stages of diabetic mice.^30^^,^^37^ Despite these ambiguous data on its mode of action, a strong body of experimental and epidemiological evidence showed that both circulating and plaque levels of SPP1 are associated with plaque inflammation, instability and cardiovascular events,^38^ suggesting that combined effects of PHD2cko on macrophage apoptosis and fibroblasts may aggravate atherosclerosis. Interestingly, SPP1 is known for its anti-apoptotic properties in numerous cell types.^39^^,^^40^ One could speculate that anti-apoptotic macrophage signalling via SPP1 would lead to paracrine, pro-fibrotic signalling. Interestingly, pro-apoptotic BNIP3 mRNA was enhanced across all macrophage subsets, while SPP1 up-regulation was most pronounced in TREM2hi macrophages. This suggests a possible new function of TREM2hi macrophages, in addition to their putative role related to lipid uptake, lysosomal digestion and lower inflammatory profile.^21^ Interestingly, TREM2hi plaque macrophages express high levels of MMPs, suggesting matrix degradation capacity. Yet, our data suggest a pro-fibrotic potential of TREM2hi plaque macrophages, consistent with fibrosis-association of TREM2+ liver macrophages,^41^ and suggested for human plaques.^42^ Our findings underline the important and unique insights derived from studying cell type subsets at the single-cell level. Further studies are required to substantiate this observation, such as measuring SPP1 protein levels in sorted macrophage subsets with sufficient cell numbers and/or bulk RNA sequencing of all WT and PD2cko macrophage subsets. To fully support the impact of SPP1 in the entire TREM2hi macrophages in PHD2cko plaques, loss of function experiments are also warranted. As expression of the TREM2 gene itself is unaltered in our study, targeting only trem2 would not be sufficient. Unfortunately, it is unclear what (epi)genetic factors are driving the TREM2hi macrophage subset at the moment. Therefore, causality experiments with a loss-of-function strategy aimed at the entire subset are not yet feasible. It is important to provide context of our study on the impact of hypoxia, and HIF-dependent and HIF-independent effects of PHDs on atherogenesis. In general, hypoxia and transient HIF activation are known to drive fibrosis and tissue repair, however, chronic hypoxia can also lead to excessive scar formation in lung and liver.^42^^,^^43^ In the context of atherosclerosis we show here that PHD2cko and exacerbated hypoxic signalling also leads to excessive fibrosis in plaques. Chronic application of environmental hypoxia has also been linked to aggravated plaque development in ApoE ko mice, but fibrosis was not measured.^44^ Reversal of hypoxia by admission of high oxygen gas, carbogen, in Ldlr ko mice did not affect collagen accumulation or induction of apoptosis, but improved efferocytosis and necrotic core formation.^2^ Moreover, two studies reporting on HIF1α-ko bone marrow transplantations show either no effect on atherogenesis, or ameliorated plaque size, necrosis and apoptosis, while fibrosis and macrophage content was not changed.^7^^,^^46^ This underscores divergence of cell-specific and systemic effects, and the possibility of HIF-independent effects of PHDs. More cell-specific effects of hypoxia can be seen in regards to apoptosis. In neutrophils of normocholesterolaemic mice, hypoxia led to a decrease of pro-apoptotic proteins, while in macrophages hypoxia induced apoptosis, and HIF1 deficiency reduced apoptosis.^7^ Strikingly both effects are achieved via NFκB signalling.^47^^,^^48^ Lastly, PHD1 and 2 whole-body KO mice showed strong amelioration of hypercholesterolaemia, while the effect of PHD3 stromal cell is not yet known.^10^^,^^11^ As PHD1, 2, and 3 myeloid deficiency did not alter cholesterol levels, this suggests that stromal cells are responsible for PHD-dependent effects on cholesterol biology. Indeed, intestine and liver were proposed to govern these responses in PHD1ko and PHD2ko mice respectively, although organ-specific PHDko would be needed to fully prove this. At the very least, the current study shows that PHD1 myeloid cells do not affect vascular biology in hyperlipidaemic conditions. When taken together, this emphasizes the complexity of cell-type specific, temporal, and lipidaemia-related reactions to low oxygen tensions and HIF signalling in the vessel wall.

The presence of both destabilizing, pro-apoptotic vs. stabilizing, pro-fibrotic effects in our PHD2cko model depict the complex role of hypoxia and/or HIF signalling in plaque development. It can be argued that larger plaques are never a positive feature. However, one could claim that fibrosis significantly increases plaque stability. When extrapolating these findings to humans, that would mean decreased possibility of plaque rupture and thus clinical manifestations. Currently, various PHD inhibitors are approved for treatment of anaemia upon chronic kidney disease. PHD inhibition in mice and man unexpectedly lowered cholesterol, as was shown by our group and others.^11^^,^^49^^,^^50^ Notably, most inhibitors show PHD2 selectivity,^12^^,^^51^ and treatment of up to 4 years is potentially long enough to affect plaque phenotype. Our current study shows development of both beneficial, and detrimental plaque traits related to PHD2 inhibition. These aspects could be taken into account when targeting PHD inhibition in humans.

The current comparison between PHD isoforms is limited as we made use of two different models to assess the effects of PHD knock-down (KD) in myeloid cells: cell type conditional LysMcre-mediated knockdown of PHD2, and bone marrow transplantations of PHD1 and PHD3. Both models are however well-established models to assess myeloid-specific KD effects. Irradiation could have influenced smooth muscle cell contribution to plaque formation and fibrosis, as suggested by Newman *et al*.^51^ Thus, we cannot exclude that plaque composition could have been influenced in our PHD1 and PHD3 knock-out models, abolishing or enhancing possible fibrotic effects.

Another limitation of our models is that PHDs were silenced in both macrophages and neutrophils, while our current study mainly focused on macrophages. However, neutrophil numbers were unchanged both in the blood and in our single-cell RNA seq dataset of PHD2 WT and cko plaques, and similar neutrophil content was observed in PHD3Ko plaques. In addition, neither BNIP3 nor SPP1 were significantly up-regulated in neutrophils of PHD2cko plaques analysed by single-cell sequencing. Hence, this supports that macrophages are predominantly responsible for the observed plaque phenotypes.

To conclude, we show that myeloid PHD isoforms have important and differing roles in atherosclerosis. Myeloid PHD2cko and PHD3ko, but not PHD1ko, lead to an exaggerated HIF-BNIP3 apoptotic pathway in macrophages. In addition, paracrine signalling in PHD2cKO mice enhanced collagen secretion by fibroblasts.

## Supplementary material

Supplementary material is available at *Cardiovascular Research* online.

## Authors’ contributions

K.v.K., J.A.F.D., T.L.T., and J.C.S. conceived and designed the study. K.v.K., J.A.F.D., T.L.T., E.M., J.d.B., and H.J. performed experiments and/or analysed data. J.P.P. and J.S.R. performed the bioinformatic analysis. C.K. and R.K. provided the 10× Genomics single-cell V2 reagents kit and fluidics machine. P.C. provided the PHD mouse models. M.G. performed pathological analysis on murine plaques. Human BiKE cohort design and acquisition was done by L.M. and U.H., MaasHPS cohort design and acquisition was done by B.M.E.M., J.C.S., and E.A.L.B. C.P.M.R. and L.S. provided AnxA5-FP488 for apoptosis detection *in vitro*. J.A.F.D., K.v.K., and J.C.S. wrote the main manuscript text. A.H.B. provided experimental advice on editing of the manuscript. K.v.K., J.A.F.D., J.P.P., T.L.T., and J.C.S. prepared the figures. All authors reviewed and approved the manuscript.

**Conflict of interest:** J.C.S. reports grants from the Dutch Organization for scientific research (016.116.017 VENI fellowship and VIDI fellowship 0.16.186.364), grants from Dr. Dekker senior postdoc fellowship of the Dutch Heart Foundation 2016T060, and the Fondation Leducq during the conduct of the study. L.S. reports grants from Bayer, grants from Boehringer Ingelheim, grants from NattoPharma, grants from Immunodiagnostics systems (IDS), outside the submitted work. J.S.R. reports grants from GSK, grants from Sanofi, personal fees from Travere Therapeutics, outside the submitted work. R.K. reports grants from Chugai, outside the submitted work. Authors were not involved in research design, execution, analysis, interpretation, and reporting.

## Funding

This work was supported by the VENI and VIDI fellowship of the Dutch Organization for scientific research (to J.C.S. 016.116.017, 0.16.186.364), a Dr. Dekker senior postdoc fellowship of the Dutch Heart Foundation (to J.C.S., 2016T060), a Fondation Leducq transatlantic network of excellence (Autophagy in 15CVD04 to J.C.S.) and two CARIM PhD fellowships (to T.L.T. 2010, and HS BAFTA for ‘talented future PhD candidates’ to J.A.F.D., 2018), and the JRC for Computational Biomedicine (JSR), which is partially funded by Bayer AG. A.H.B. is supported by the British Heart Foundation Chair of Translational Cardiovascular Sciences.

## Data availability

Data relating to this article can be found in the article itself or online supplementary material. Reproducible code for the transcriptomics analysis is accessible at the GitHub repository: https://github.com/saezlab/Myeloid_PHD2_atherogenesis.

Raw sequencing data and processed matrices of gene expression from RNA-seq and single-cell RNAseq are available at Gene Expression Omnibus, and can be accessed with GSE150090.

## Supplementary Material

cvab152_Supplementary_Data

## Notes

Translational perspective This study shows that myeloid prolyl hydroxylase (PHD) isoforms PHD2 and PHD3 worsen plaque characteristics and phenotype, such as plaque size, macrophage accumulation, apoptosis, and collagen accumulation in mice. We show both direct effects on macrophages and paracrine effects of macrophage PHD2 loss on vessel wall fibroblast populations. Broad spectrum-PHD inhibitors, e.g. Roxadustat, are currently being prescribed to chronic kidney disease patients, who are already at risk for cardiovascular disease. When considering this study and the pro-fibrotic and pro-apoptotic effects we report, broad PHD inhibition may therefore be sub-optimal and more targeted PHD inhibition of PHD1 should be considered.
