# Supplementary material for: Deficiency of myeloid PHD proteins aggravates atherogenesis via macrophage apoptosis and paracrine fibrotic signalling
Source: Cardiovasc Res. 2021 Apr 29;118(5):1232–46. doi: 10.1093/cvr/cvab152 (PMC8953448; doi:10.1093/cvr/cvab152)
Supplement: cvab152_Supplementary_Data [file cvab152_supplementary_data.pdf]

Online data supplement related to:

DEFICIENCY OF MYELOID PHD PROTEINS AGGRAVATES ATHEROGENESIS VIA MACROPHAGE APOPTOSIS AND PARACRINE FIBROTIC SIGNALING

Atherogenic effects of myeloid PHD knockdown

van Kuijk K[a,b]†, Demandt JAF, MD, PhD,[a,b]†, Perales-Patón J, PhD, [c,d,o]†, Theelen TL, PhD, [a,b], Kuppe C, MD, PhD, [d], Marsch E, PhD, [a,b], de Bruijn J, MD, [a,b], Jin H[a,b], Gijbels MJ, PhD, [a,b,e,f,g], Matic L, PhD, [h], Mees BME, MD, PhD, [a,i], Reutelingsperger CPM, PhD, [a,j], Hedin U, MD, PhD, [h], Biessen EAL, PhD, [a,b,k], Carmeliet P, MD, PhD, [l], Baker AH, PhD, [a,m], Kramann RK, PhD, [d,n], Schurgers LJ, PhD, [a,d,j], Saez-Rodriguez J, PhD[c,o], Sluimer JC, PhD, *[a,b,m]

Table of content:

Supplementary Figure S1-11

Supplementary Tables S1-8

Supplementary methods

References 53-70

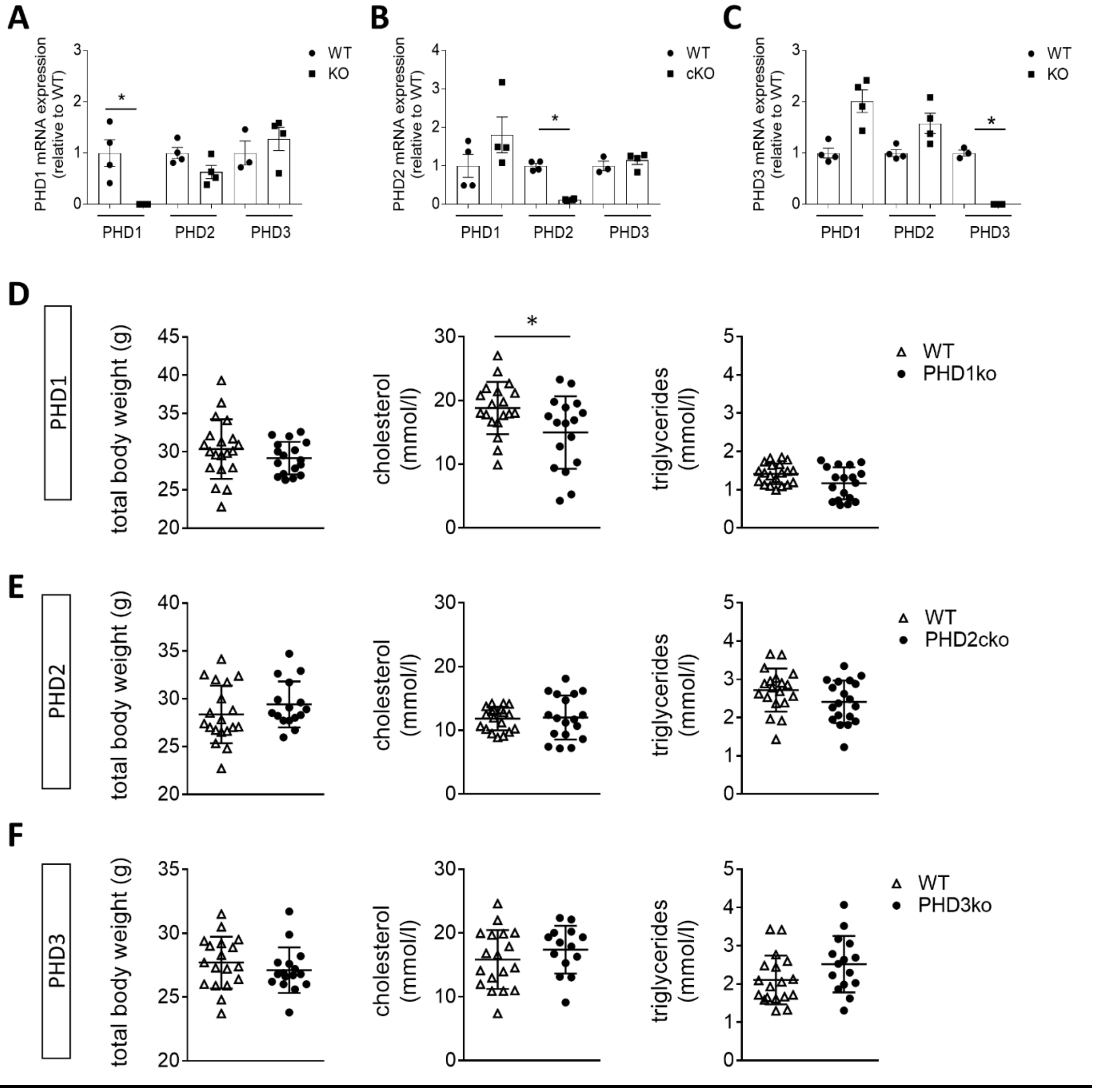

Supplementary Figure S1. Knockdown confirmation and circulating lipids in PHD1ko, PHD2cko and PHD3ko mice (**A**) Expression of PHD1, (**B**) PHD2, and (**C**) PHD3 mRNA in PHD1ko, PHD2cko and PHD3ko BMDM compared to respective controls. (**D**) Total body weight, circulating cholesterol and triglycerides of PHD1ko, (**E**) PHD2cko and (**F**) PHD3ko mice and respective controls (n=16-20 per group). Statistical analyses were performed using a two-way ANOVA, with Bonferroni post-hoc test (A-C) or a student t-test (D-F). All results show mean ± SEM. *P<0.05.

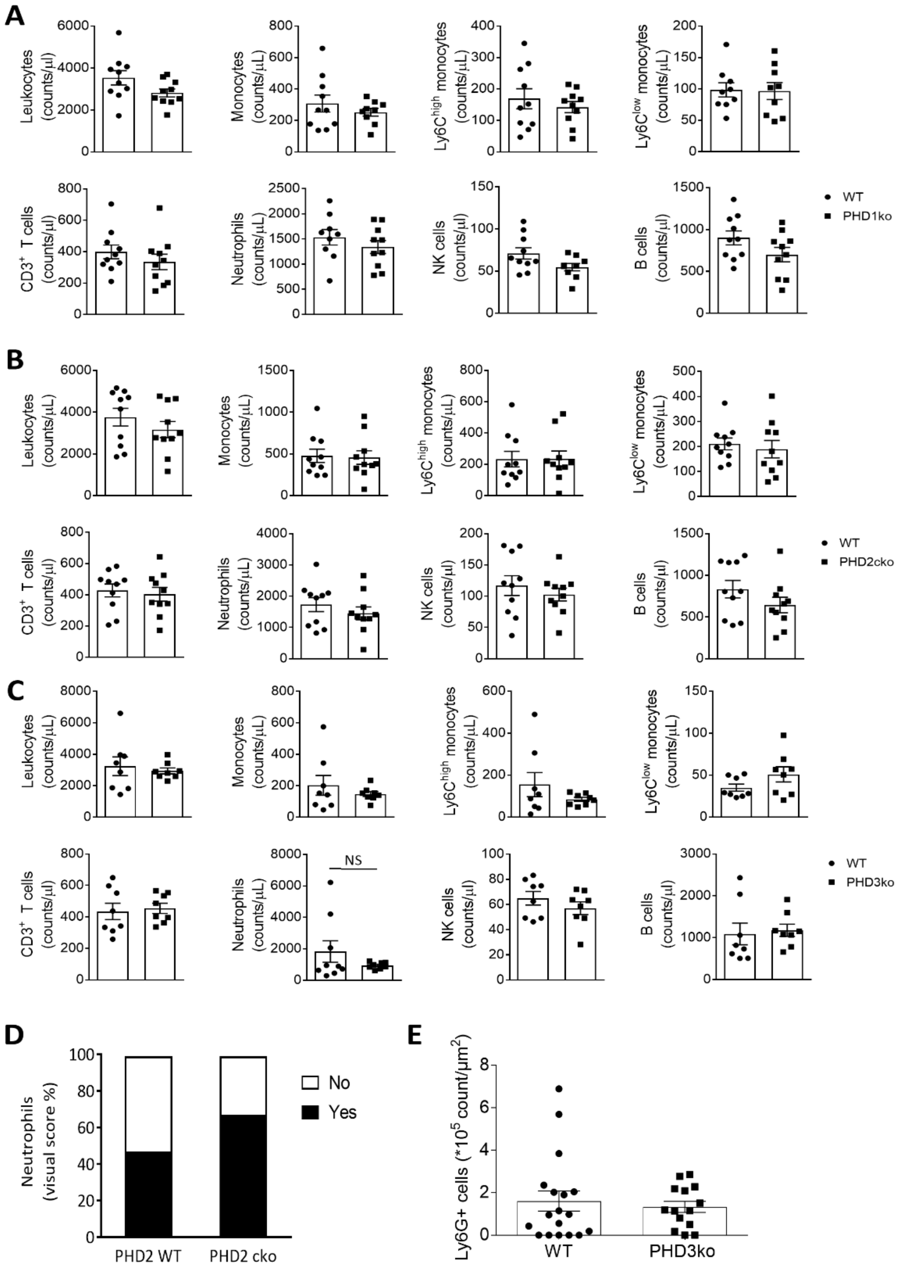

Supplementary Figure S2. No change in circulating leukocyte subsets in myeloid PHD knockout mice and respective controls (**A**) Circulating leukocyte subsets of PHD1ko, (**B**) PHD2cko, and (**C**) PHD3ko mice (circles) and respective controls (squares, n=20 per group for PHD1ko and PHD2cko, n=16-18 per group for PHD3ko). (**D**) Neutrophils presence scored as present (Yes) vs. not present (No) by animal pathologist MG. (**E**) Quantification of Ly6G positive neutrophils in PHD3ko and corresponding WT plaques, N=18 per group. Statistical analyses were performed using a student t-test (A-C, E). All results show mean ± SEM.

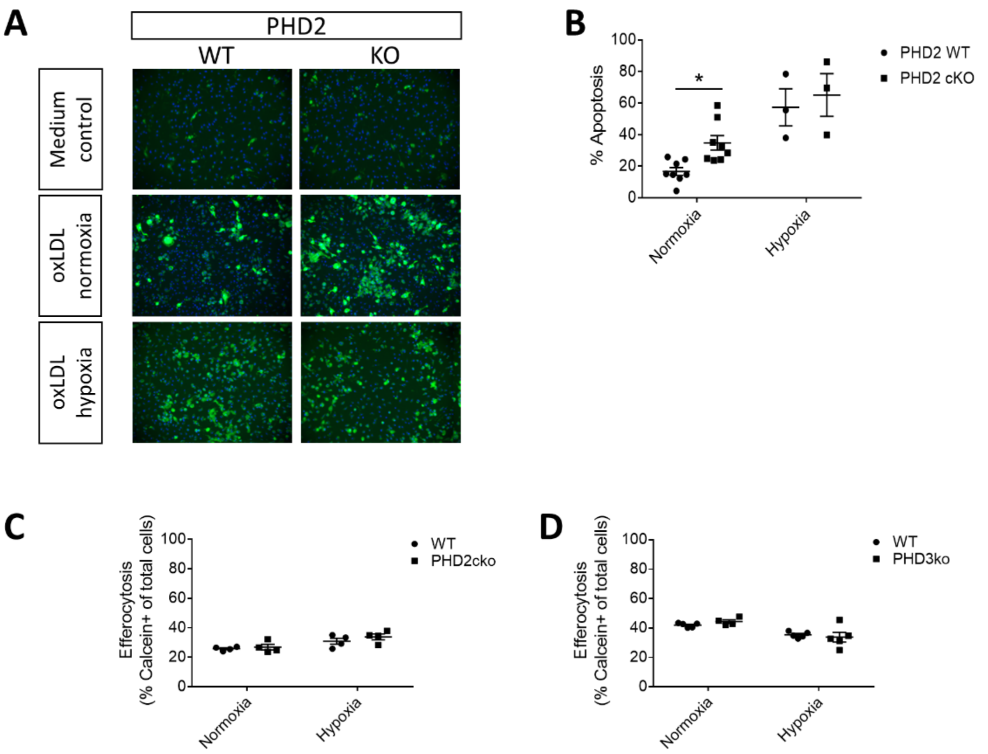

Supplementary Figure S3. Unchanged efferocytosis in PHD2cko and PHD3ko BMDMs (**A**) Representative images and (**B**) quantifications of *in vitro* Wt and PHD2cko BMDM apoptosis induced by oxLDL in normoxic and hypoxic conditions. (**C-D**) *In vitro* BMDM efferocytosis under normoxic and hypoxic conditions. Expressed as the percentage of Calcein positive BMDMs. Statistical analyses were performed using a two-way ANOVA, with Bonferroni post-hoc test (B-D). All results show mean ± SEM.

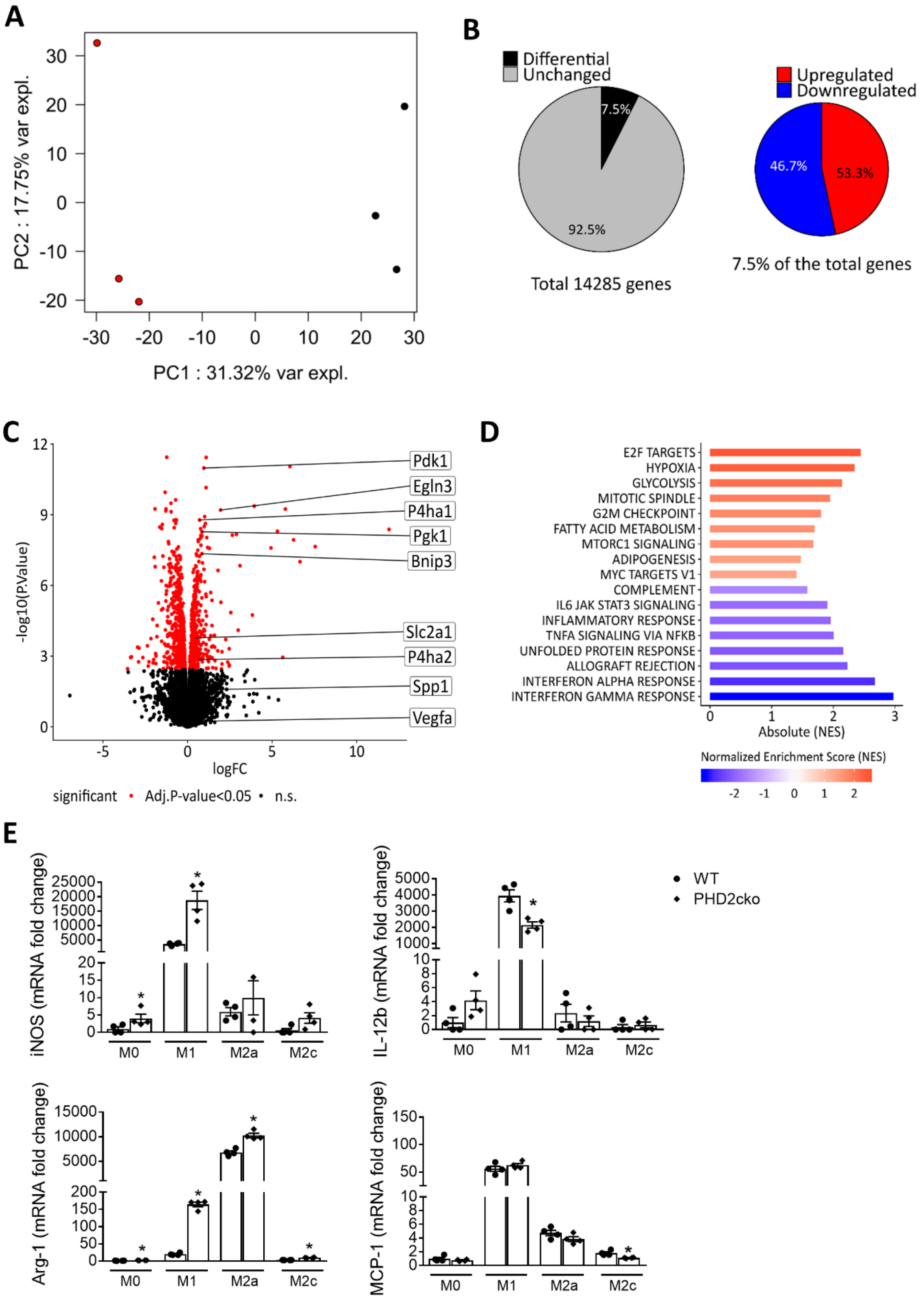

Supplementary Figure S4. Transcriptomic analysis of PHD2cko and WT BMDMs in vitro (**A**) Principal component analysis depicting separation of samples depending on genotype. Black: WT. Red: PHD2cko. (**B**) Pie chart showing number of differentially up and down regulated genes in BMDM. (**C**) Volcano plot showing significant differentially expressed genes between WT and PHD2cko

BMDMs in red, with genes relevant for hypoxia signaling and apoptosis marked. Triplicate samples for RNAseq in each group. (**D**) Barplot showing the Normalized Enrichment Scores (NES) for the statistically significant hallmark gene sets enriched in BMDM PHD2cko as compared to WT from the pre-ranked GSEA. (**E**) Expression of macrophage polarization genes in PHD2cko and corresponding WT bone marrow derived macrophages measured by qRT-PCR. All polarization stimuli were given for 24 hours. M1 received IFNγ (100u/ml) and LPS (10ng/ml), M2A received IL-4 (20ng/ml) and M2C received IL-10 (10ng/ml). Statistical analyses were performed using a two-way ANOVA, with Bonferroni post-hoc test (E). All results show mean ± SEM. *P<0.05.

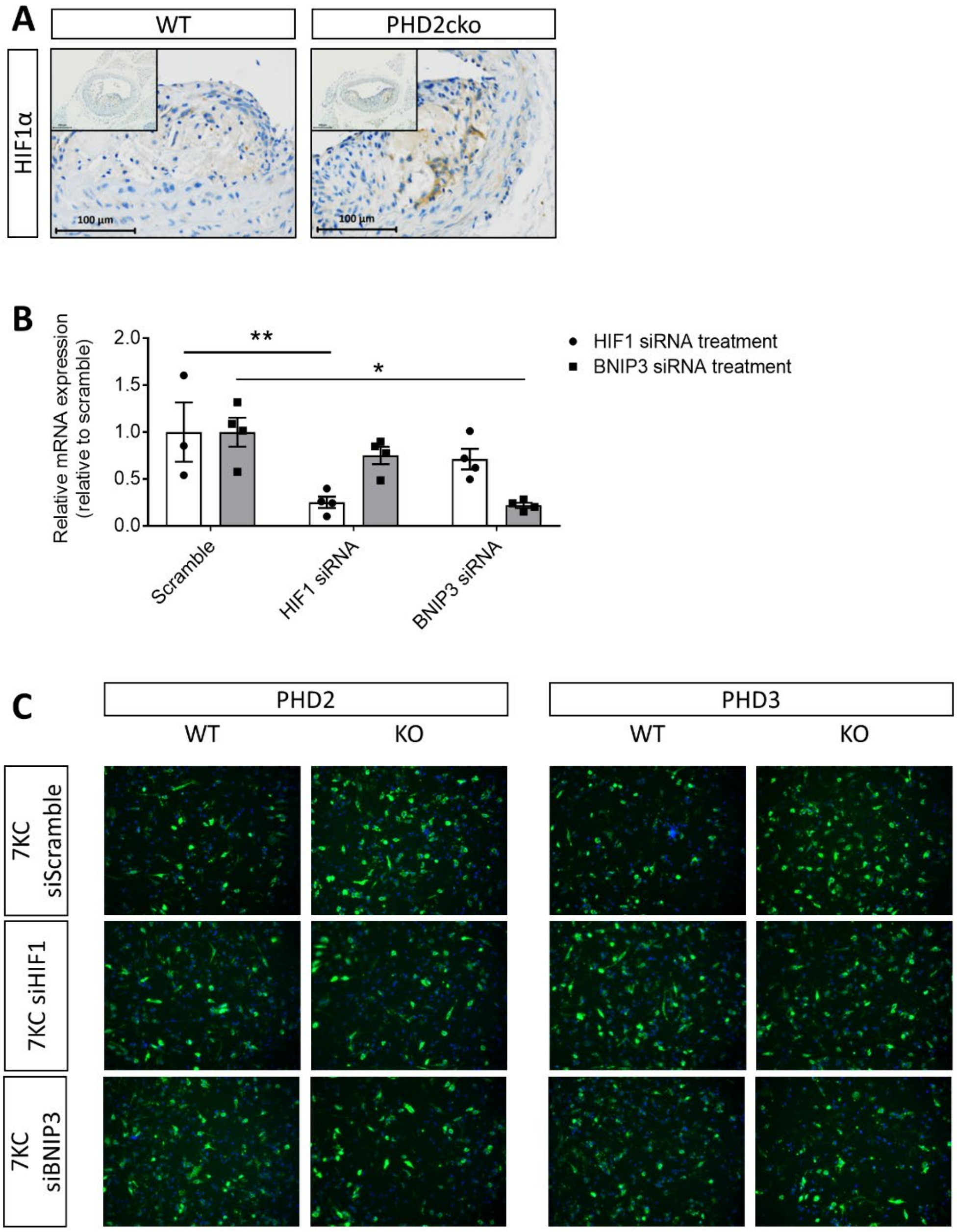

Supplementary Figure S5. HIF1α and BNIP3 expression and silencing (**A**) Representative microphotograph of HIF1 stained plaque in murine WT and PHD2cko brachiocephalic artery. Scalebar 100µm. (**B**) Expression of HIF1α (white bars) and BNIP3 mRNA (grey bars) upon silencing with respective siRNA on y-axis compared to scramble siRNA. (**C**) Representative microphotographs of in vitro apoptosis measured by ANX5-FP488 induced by 7KC in control, PHD2cko and PHD3ko after transfection with scramble (SCR), HIF1α, and BNIP3 siRNA. Statistical analyses were performed using a two-way ANOVA, with Bonferroni post-hoc test (B). All results show mean ± SEM.

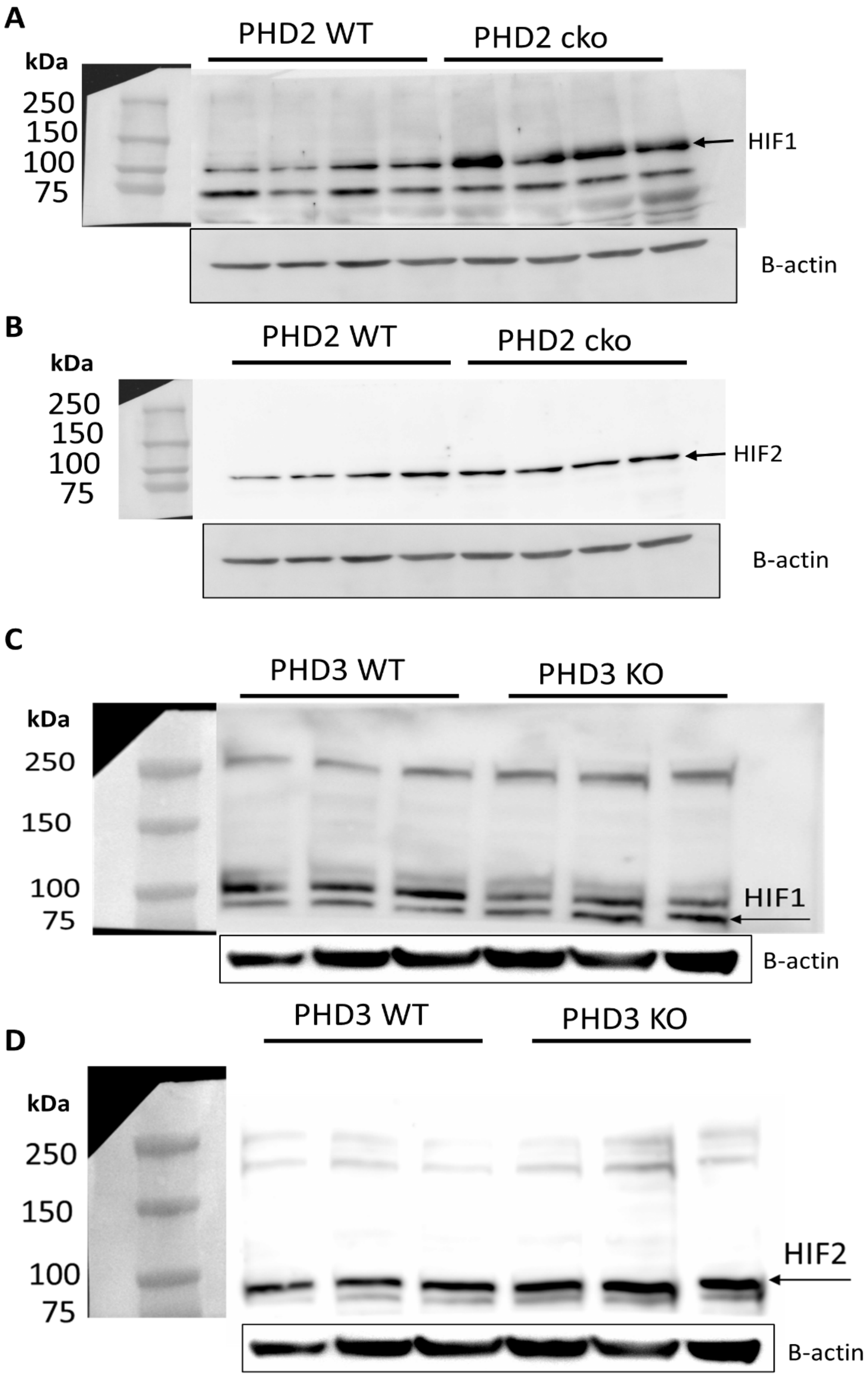

Supplementary Figure S6. Full western blots for figures 3F-G. PHD2 WT vs cko protein expression of HIF1 (**A**) and HIF2 (**B**). PHD3 WT vs. ko protein expression of HIF1 (**C**) and HIF2 (**D**). All with corresponding b-actin control. Blots were cut at 75kDa.

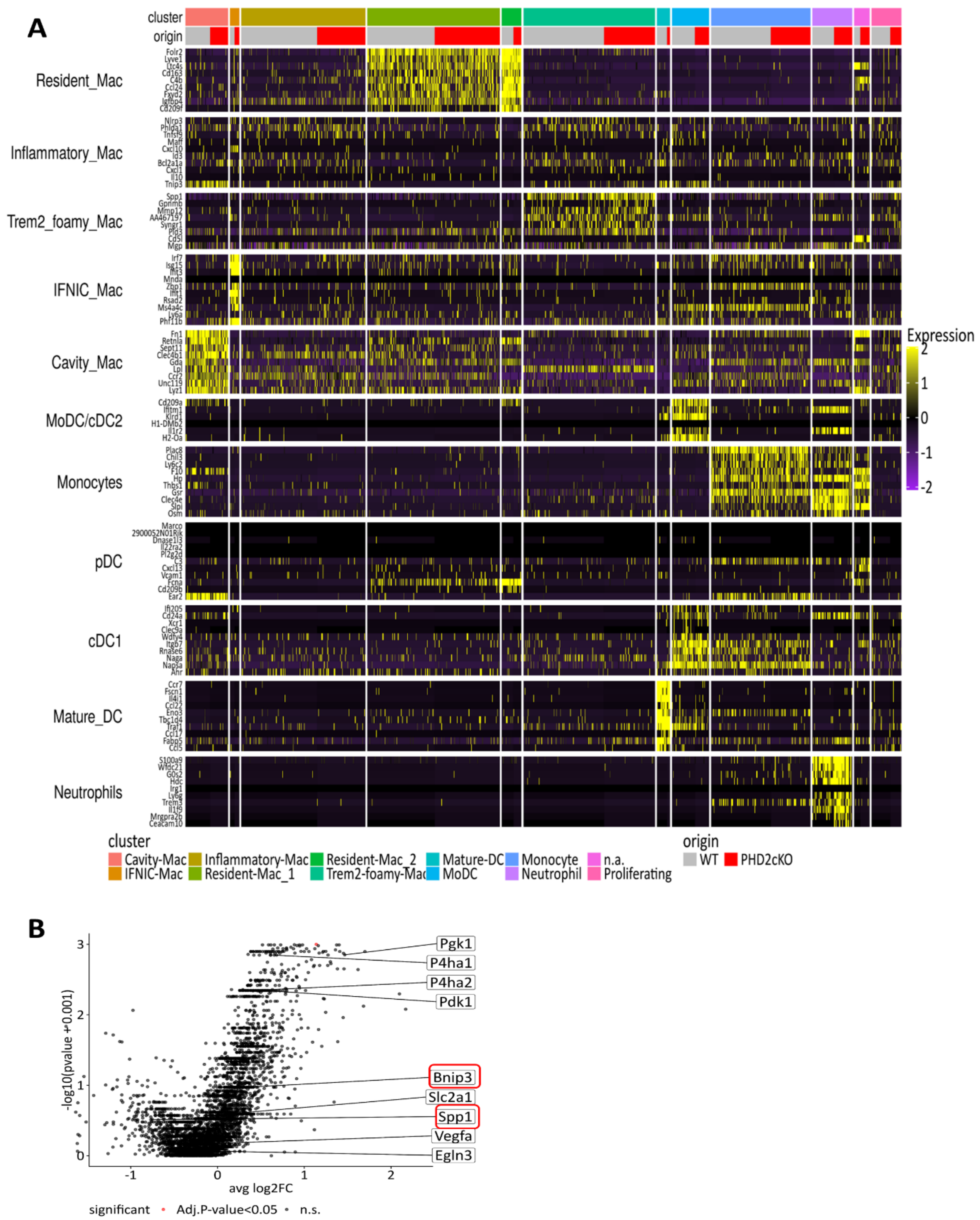

Supplementary Figure S7. Cell annotation of myeloid leukocytes in single cell sequencing dataset from PHD2 WT and cko plaques. (**A**) Heatmap depicting cell type annotation based on the top 10 markers reported from Zernecke et al. [53]. Rows are genes. Columns are cells. Columns were grouped by the outcome from unsupervised clustering (12 clusters). Gene-level expression was scaled across cells. (**B**) Volcano plot depicting differentially expressed genes in high PHD2cko signature neutrophils (n=7 cells) vs. low PHD2cko signature neutrophils (n=30 cells).

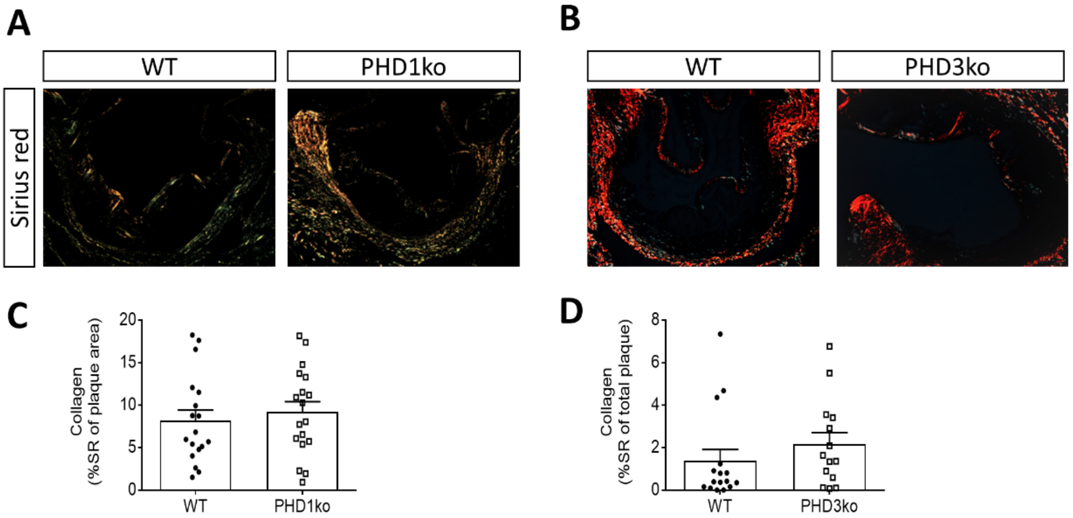

Supplementary Figure S8. Myeloid PHD1ko and PHD3ko did not affect plaque collagen (**A**) Representative microphotographs of Sirius red stained aortic root sections of PHD1ko, and (**B**) PHD3ko mice and respective controls. (**C**) Quantification of Sirius red positive area of PHD1ko and (**D**) PHD3ko mice and their controls in 16-20 mice per group. Statistical analyses were performed using a student t-test (C-D). All results show mean ± SEM.

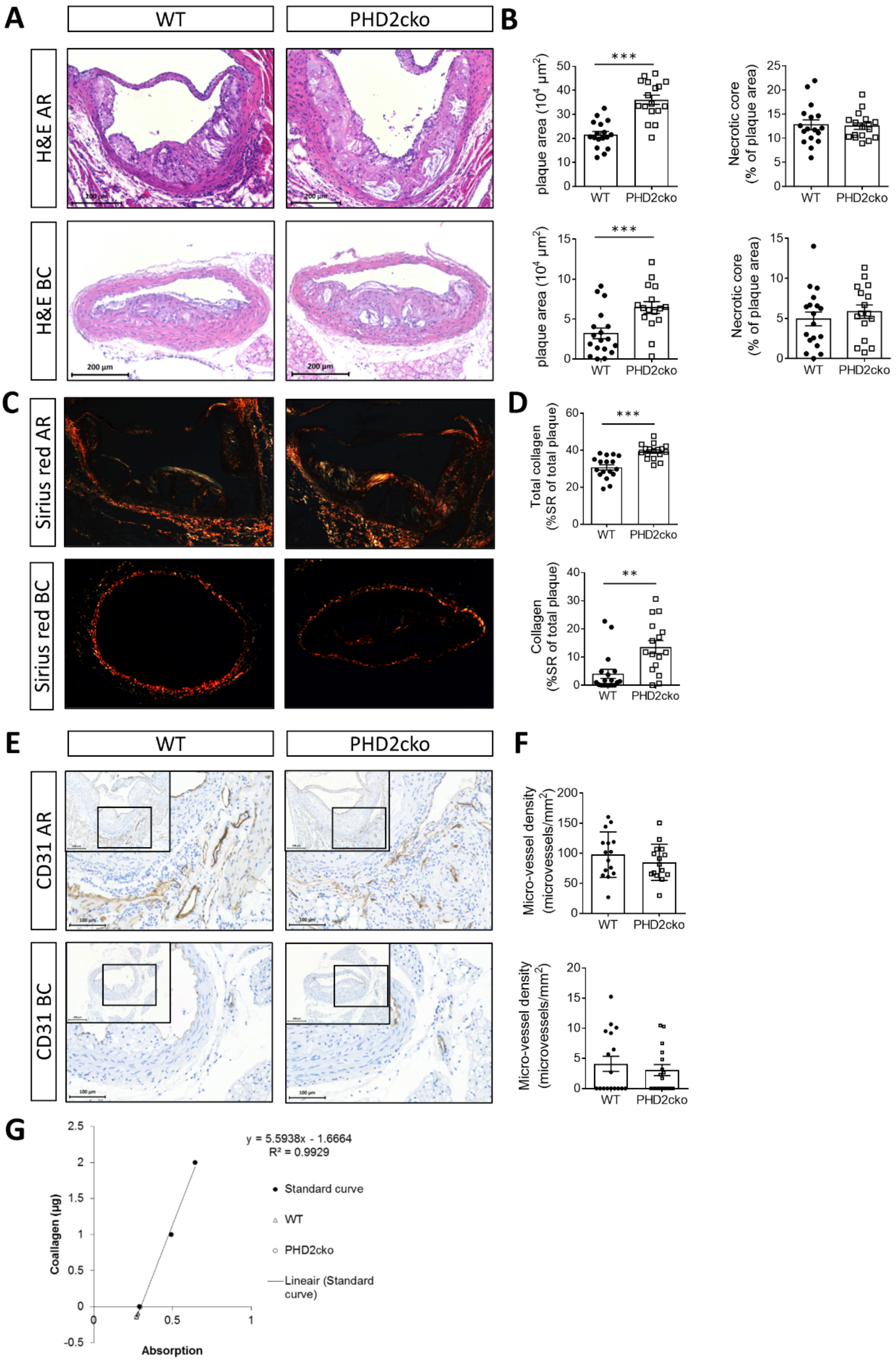

Supplementary Figure S9. Increased plaque size in PHD2cko mice is preserved in 12 weeks HCD diet fed mice, and two vascular beds (**A**) Representative microphotographs of aortic root lesions stained with HE in PHD2cko and WT control mice fed a 12-week high-cholesterol diet. (**B**)

Quantification of plaque size and necrotic content. (**C**) Sirius red stained aortic root and brachiocephalic (BC) artery sections of WT and PHD2cko mice fed a 12-week HCD. (**E**) Representative microphotographs of CD31 stained aortic root and brachiocephalic sections of PHD2cko and WT control mice. (**F**) Quantification of micro-vessel density in adventitia of aortic root and brachiocephalic artery. All groups contain 16-20 mice. Statistical analyses were performed using a student t-test (B, D, F). All results show mean ± SEM. ***$P<0.001$. Scale bar HE 200µm, CD31 100µm. (**G**) Absorption and collagen of BMDM supernatant and standard curve samples.

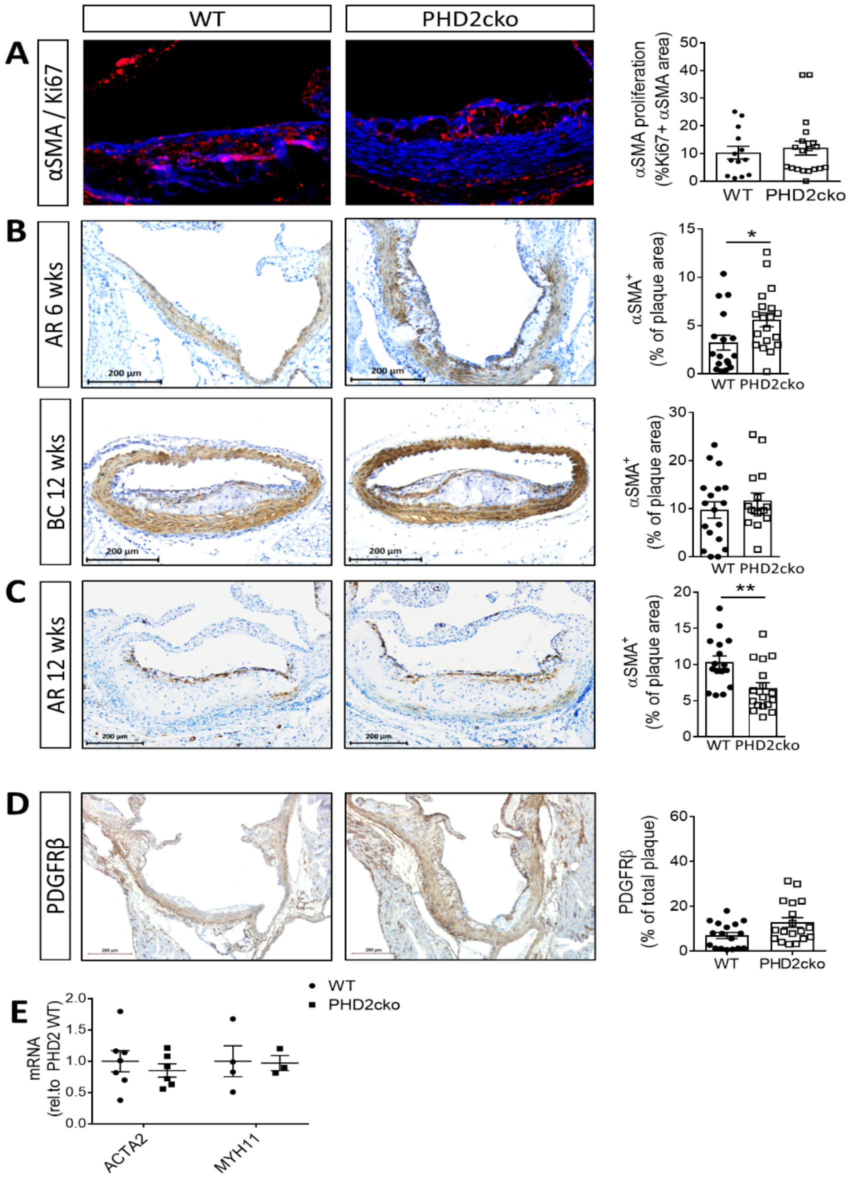

Supplementary Figure S10. Plaque content of αSMA in 6 and 12 week HCD diet fed PHD2cko mice, and two vascular beds (**A**) Pseudo-fluorescent image of αSMA/Ki67 (red/blue, respectively) staining of mesenchymal (MC) proliferation in the aortic root plaque. Magnification 400x. (**B**) Representative microphotographs and quantification of plaque αSMA content in PHD2cko and WT control mice of aortic roots fed a 6-week high-cholesterol diet, and (**C**). In brachiocephalic and aortic root of mice fed a 12-week high-cholesterol diet. All groups contain 16-20 mice. (**D**) Representative microphotographs and quantification of PDGFRβ (brown precipitate, DAB) in aortic root of mice fed a six-week high-cholesterol diet. Scalebar 100μm. (**E**) mRNA expression of myogenic genes in 3T3 fibroblasts incubated in PHD2cko BMDM conditioned medium for 72 hours. Statistical analyses were performed using a student t-test (A-D) or two-way ANOVA with Bonferroni post-hoc test (E). All results show mean ± SEM. *P<0.05 **P<0.01. Scale bar 200μm.

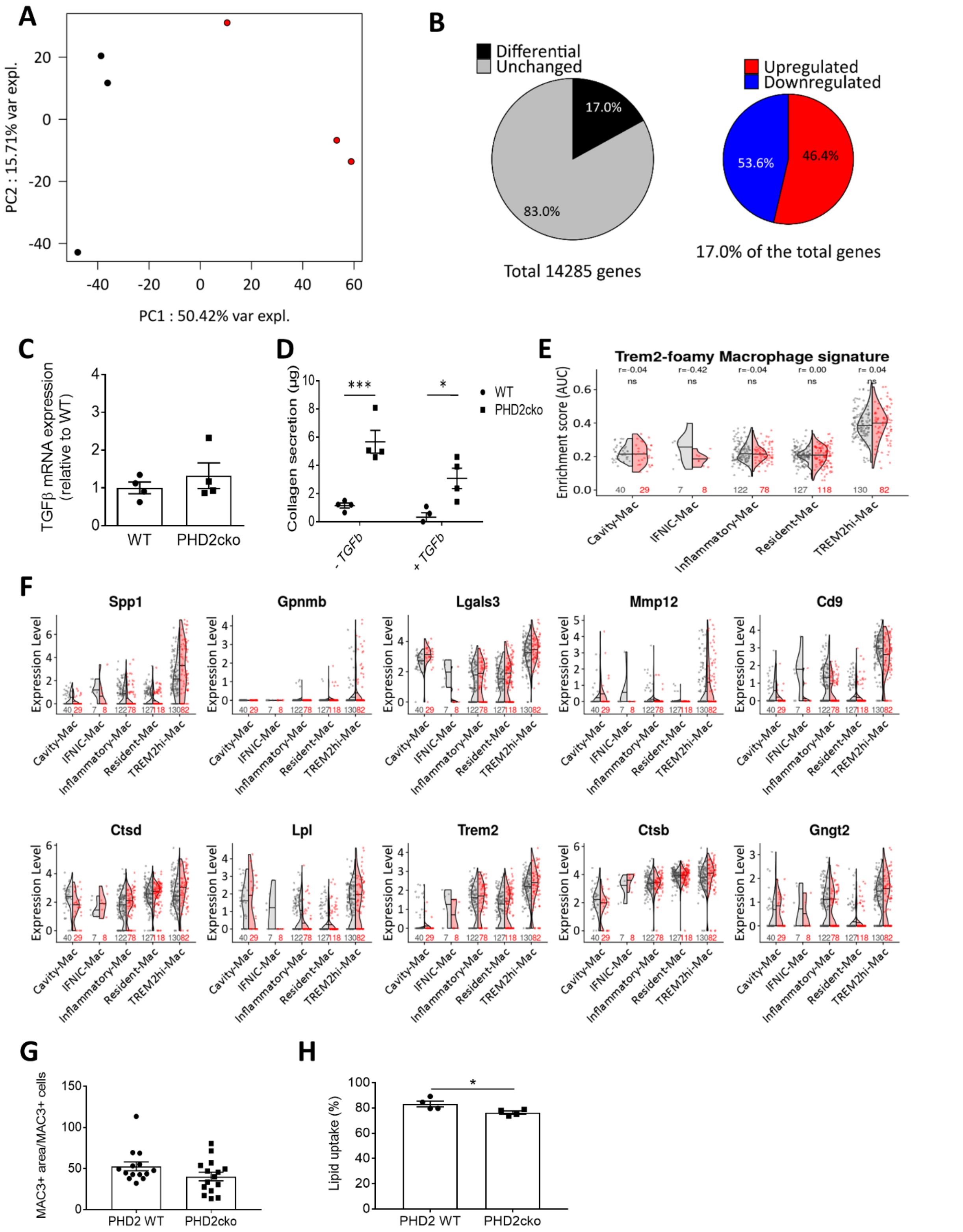

Supplementary Figure S11. Transcriptomic analysis of fibroblasts incubated in medium conditioned by PHD2cko and WT BMDM and *in vitro* stimulation fibroblasts with TGF-β

(**A**)Principal component analysis depicting separation of samples depending on exposure. Black: WT-conditioned medium, Red: PHD2cko-conditioned medium. (**B**) Pie chart showing number of up and

down regulated genes in fibroblasts. Triplicate samples for RNAseq in each group. (**C**) In vitro mRNA expression of TGFβ1 by WT and PHD2cko BMDM was similar. (**D**) Addition of TGFβ to fibroblasts did not increase collagen secretion of fibroblasts incubated with WT-conditioned medium to the same level as PHD2cko-contioned medium. (**E**) Split violin plot of PHD2 WT (grey) and cko (red) macrophages showing the single-cell enrichment score of the TREM2hi signature (TREM2hi macrophage DEGs from Zernecke et al. 2020). (**F**) Individual gene expression patterns of the top 10 differentially over-expressed genes from the TREM2hi signature in PHD2 WT and cko macrophages. (**G**) MAC3+ area divided by MAC3+ cells in PHD2 WT and cko plaques of 6 weeks high cholesterol diet study. (**H**) Lipid uptake of fluorescently labeled cholesterol in PHD2 WT and cko bone marrow derived macrophages. Statistical analyses were performed using a student t-test (C) or two-way ANOVA with Bonferroni post-hoc test (D). All results show mean ± SEM. *P<0.05 ***P<0.001.

SUPPLEMENTARY TABLES

Supplementary Table S1. Primer sequences used for real-time quantitative PCR

Gene	forward primer (5'-3')	reverse primer (5'-3')
**18S**	GTA ACC CGT TGA ACC CCA TT	CCA TCC AAT CGG TAG TAG CG
**Cyclo**	CAA ATG CTG GAC CAA ACA CAA	AGT TAG CAT GCC AGA GTC TCG TT
**PHD1**	CAT CAA TGG GCG CAC CA	GAT TGT CAA CAT GCC TCA CGT AC
**PHD2**	TAA ACG GCC GAA CGA AAG C	GGG TTA TCA ACG TGA CGG ACA
**PHD3**	CTA TGT CAA GGA GCG GTC CAA	GTC CAC ATG GCG AAC ATA ACC
**BNIP3**	CCT GTC GCA GTT GGG TTC	GAA GTG CAG TTC TAC CCA GGA G
**HIF1α**	TGC TCA TCA GTT GCC ACT TC	CCA TCT GTG CCT TCA TCT CA
**TGFβ**	GCC CTT CCT GCT CCT CAT G	CCG CAC ACA GCA GTT CTT CTC
**ACTA2**	GAC TAC TGC CGA GCG TGA G	ATA GGT GGT TTC GTG GAT GC
**MYH11**	TGG ACA CCA TGT CAG GGA AA	ATG GAC ACA AGT GCT AAG CAG TCT

Supplementary Table S2. Pearson correlation of PHD3 mRNA expression with CD68, HIF1 and HIF2 mRNA expression in plaques of BiKE cohort subjects (n=127)

	**PHD1**		**PHD2**		**PHD3**	
	Correlation	p-value	Correlation	p value	Correlation	p value
**CD68**	-0.17	0.064	0.42	< 0.001	0.64	< 0.001
**HIF1**	-0.28	0.002	0.44	< 0.001	0.43	< 0.001
**HIF2**	0.19	0.032	-0.18	0.047	-0.28	0.002

Supplementary Table S3. PROGENy pathway analysis of differentially expressed genes between WT and PDH2cko BMDM

	Activity (Z-score)	P-value
**HYPOXIA**	14.85	$<1.0 * 10^{-4}$
**MAPK**	1.84	$6.6*10^{-2}$
**TGFB**	0.82	$4.1 * 10^{-1}$
**EGFR**	0.72	$4.7 * 10^{-1}$
**P53**	-0.96	$3.4 * 10^{-1}$
**TRAIL**	-1.27	$2.0 * 10^{-1}$
**ESTROGEN**	-1.30	$1.9 * 10^{-1}$
**WNT**	-1.31	$1.9 * 10^{-1}$
**ANDROGEN**	-1.84	$6.6 * 10^{-2}$
**VEGF**	-2.39	$1.7 * 10^{-2}$
**PI3K**	-3.14	$1.7 * 10^{-3}$
**TNFA**	-3.74	$1.8 * 10^{-4}$
**NFKB**	-6.22	$<1.0 * 10^{-4}$
**JAK.STAT**	-11.07	$<1.0 * 10^{-4}$

Supplementary Table S4. DoRothEA Transcription Factor analysis between WT and PDH2cko BMDM

	logFC	AveExpr	t	P-Value	adj.PVal	B
**RFX5**	-0.48	-0.94	-6.329	0.0001	0.0051	2.220
**FOXM1**	0.30	1.75	6.186	0.0001	0.0051	2.024
**HIF1A**	0.51	2.82	6.096	0.0001	0.0051	1.899
**RFXANK**	-0.35	-0.84	-6.009	0.0001	0.0051	1.776
**RFXAP**	-0.35	-0.84	-6.009	0.0001	0.0051	1.776
**BCL3**	-0.21	4.60	-4.845	0.0005	0.0247	0.033
**IRF1**	-0.61	-0.96	-4.702	0.0007	0.0266	-0.196
**GLI2**	0.29	-0.78	4.333	0.0012	0.0426	-0.798

Only differentially active transcription factors are shown (FDR-adjusted p-value < 0.05). LogFC: estimate of the log2-fold-change Transcription factor activity corresponding to the effect or contrast. AveExpr: average log2-expression. t: moderated t-statistic. P-Value: raw p-value. adj.PVal, adjusted p-value (FDR). B: log-odds that the gene is differentially expressed

Supplemental Table S5. PHD2cko gene signature, retrieved from Bulk RNA sequencing data of PHD2 WT vs. cko bone marrow derived macrophages.

Genes	logFC	Avr. Expr	t	P-Value	Adj. P-value	B
**Pfkl**	1.10	6.21	25.77	3.70E-12	2.64E-08	18.46
**Pdk1**	0.96	6.20	23.67	1.05E-11	3.74E-08	17.40
**Cd5l**	1.09	3.09	20.22	7.05E-11	2.02E-07	15.42
**Hpgd**	6.06	-0.51	23.98	8.90E-12	3.74E-07	13.86
**Egln3**	1.95	3.04	16.84	6.32E-10	6.94E-07	13.11
**S1pr1**	1.01	5.93	15.95	1.21E-09	1.08E-06	12.52
**P4ha1**	0.71	7.11	15.52	1.67E-09	1.23E-06	12.15
**Fgd6**	1.06	3.73	15.08	2.36E-09	1.53E-06	11.95
**Tpi1**	0.99	8.23	14.70	3.17E-09	1.89E-06	11.43
**Narf**	0.77	5.25	14.44	3.94E-09	2.01E-06	11.34
**Pgk1**	0.85	8.54	14.07	5.31E-09	2.45E-06	10.89
**Tfrc**	0.79	5.56	13.65	7.62E-09	3.20E-06	10.69
**Ankrd37**	3.94	0.62	17.39	4.31E-10	6.84E-07	10.67
**Eno1**	0.86	8.70	13.60	7.91E-09	3.23E-06	10.47
**Folr2**	1.14	3.47	13.42	9.30E-09	3.69E-06	10.32
**Ak4**	2.65	3.20	13.69	7.33E-09	3.17E-06	10.32
**Anxa3**	0.76	6.30	13.32	1.01E-08	3.90E-06	10.23
**Col18a1**	0.62	5.36	13.14	1.18E-08	4.32E-06	10.05
**Wdfy1**	0.63	5.66	12.99	1.36E-08	4.84E-06	10.03
**Armc6**	1.30	3.72	12.25	2.67E-08	7.94E-06	9.63
**Atp6v0d2**	0.80	3.03	12.58	1.97E-08	6.53E-06	9.50
**Mif**	1.19	6.40	12.34	2.46E-08	7.65E-06	9.36
**Gapdh**	0.61	9.95	12.05	3.22E-08	9.21E-06	9.01
**Zfyve28**	0.52	3.08	11.68	4.62E-08	1.22E-05	8.64
**Abcd3**	0.54	6.01	11.54	5.32E-08	1.36E-05	8.58
**Pcdh7**	0.71	5.29	11.45	5.77E-08	1.39E-05	8.52
**Mefv**	0.67	-0.42	10.94	9.70E-08	2.07E-05	8.14
**Ets1**	0.88	5.08	10.82	1.11E-07	2.27E-05	8.08
**Rgs11**	1.80	1.08	10.13	2.31E-07	3.67E-05	7.50
**Mamdc2**	0.48	4.08	10.55	1.47E-07	2.80E-05	7.40
**Acacb**	3.10	2.04	10.56	1.45E-07	2.80E-05	7.35
**Tmem8**	0.51	4.93	10.47	1.59E-07	2.97E-05	7.29
**Dhrs9**	0.97	5.49	10.23	2.08E-07	3.45E-05	7.26
**Gpi1**	0.38	9.09	10.24	2.06E-07	3.45E-05	7.02
**Fnbp1l**	0.61	6.56	10.04	2.56E-07	3.93E-05	6.84
**Jak3**	0.57	5.81	9.95	2.82E-07	4.24E-05	6.73
**Igf2bp2**	0.76	5.32	9.61	4.14E-07	5.70E-05	6.72
**Slc6a8**	0.33	6.89	9.96	2.78E-07	4.23E-05	6.70
**Myo1e**	0.58	7.01	9.86	3.11E-07	4.54E-05	6.57
**Zfp868**	0.69	4.55	9.56	4.40E-07	5.88E-05	6.55
**Agl**	0.80	6.47	9.75	3.56E-07	5.03E-05	6.49
**Ighg2b**	5.32	-3.85	14.14	5.04E-07	2.40E-05	6.30
**Frrs1**	0.66	6.38	9.60	4.19E-07	5.70E-05	6.27
**Ldha**	0.56	8.51	9.61	4.16E-07	5.70E-05	6.27
**Tlr1**	0.66	3.71	9.37	5.48E-07	7.03E-05	6.07
**Fam214a**	0.62	4.58	9.29	6.00E-07	7.46E-05	6.04
**Lpl**	0.46	9.48	9.36	5.56E-07	7.03E-05	6.01
**Gcn1l1**	0.75	7.15	9.36	5.54E-07	7.03E-05	5.98
**Antxr2**	0.41	7.28	9.28	6.13E-07	7.48E-05	5.85
**Igkc**	4.94	-3.94	12.29	2.57E-08	7.80E-06	5.61

LogFC: estimate of the log2-fold-change gene. AveExpr: average log2-expression. t: moderated t-statistic. P-Value: raw p-value. adj.PVal, adjusted p-value (FDR). B: log-odds that the gene is differentially expressed

Supplementary Table S6. Pre-ranked GSEA pathway analysis of matrisome pathways, differentially expressed between fibroblasts incubated in WT- or PDH2cko-BMDM conditioned medium

Pathway	p-val	p-adj	ES	NES	Size	LEADING EDGE
**Collagens**	0.026	0.043	0.47	1.55	31	COL3A1, COL5A3, COL15A1, COL28A1, COL18A1, COL27A1, COL5A1, COL1A1, COL5A2
**ECM Glycoproteins**	0.029	0.043	-0.33	-1.43	115	TINAGL1, FBLN5, WISP2, CYR61, SMOC2, THBS1, NOV, IGFBP4, BMPER, MFGE8, MGP, NPNT, LTBP1, EMILIN2, CTGF, LAMA5, SMOC1, THBS2, AEBP1, SLIT2, IGFBP6, PCOLCE
**ECM Regulators**	0.234	0.281	-0.26	-1.12	139	SERPINE2, ADAMTSL5, LOX, CD109, MMP2, TIMP2, ADAMTS1, SERPINA1A, MMP14, SERPINE1, ADAMTS5, TIMP4, ITIH4, SERPINB1B, SERPINB5, PLOD3, MASP1, ADAMTS16, HYAL2, HYAL1, PAPPA2, AGT, SERPINB9, SERPINC1, SERPINB8, OGFOD1, ADAM23, P4HTM, SULF2, MMP11, ADAM11, KAZALD1, CST3, OGFOD2
**ECM-affiliated Proteins**	0.008	0.023	0.38	1.57	92	PLXNC1, SEMA7A, SDC3, CLEC4A1, PLXDC1, CLEC12A, CLEC5A, SEMA4D, CLEC4D, CLEC7A, SEMA3C, CLEC4E, C1QA, CLEC4N, OVGP1, C1QC, CLEC4A2, C1QB, CLEC4A3, PLXNA2, ANXA2, SEMA3A, PLXND1, CLEC2H, SEMA4A, FCNA, C1QTNF6, ANXA6, CLEC10A, CLEC9A, SEMA4C
**Proteoglycans**	0.372	0.372	-0.35	-1.05	22	PODNL1, OGN, HAPLN4, ASPN, BGN,

						BCAN
**Secreted Factors**	0.002	0.011	0.36	1.59	160	IGF1, ANGPTL4, CXCL14, CCL3, IGF2, CX3CL1, PDGFB, TGFB1, CCL2, TNF, CCL4, CCL12, CCL11, IL16, CCL5, EBI3, CXCL10, IL1RN, S100A1, PF4, CCL9, CCL7, CXCL2, CCL6, CXCL12, TNFSF14, CCL17, FSTL1, FGF13, FGF11, TNFSF13, S100A8, S100A13, S100A16, CLCF1, OSM

ECM: extracellular matrix; p-adj, FDR-adjusted p-value, ES, enrichment score, NES, enrichment score normalized to mean enrichment of random samples of the same size

Supplementary Table S7. PROGENy pathway analysis of differentially expressed genes of fibroblasts incubated in WT- or PDH2cko-BMDM conditioned medium

	Activity (Z-score)	P-value
**JAK.STAT**	6.60	$4.0 * 10^{-11}$
**TNFA**	4.78	$1.8 * 10^{-6}$
**NFKB**	4.37	$1.2 * 10^{-5}$
**MAPK**	4.28	$1.9 * 10^{-5}$
**Hypoxia**	3.90	$9.7 * 10^{-5}$
**EGFR**	1.53	$1.3 * 10^{-1}$
**PI3K**	0.97	$3.3 * 10^{-1}$
**WNT**	0.65	$5.1 * 10^{-1}$
**TRAIL**	-0.03	$9.7 * 10^{-1}$
**Androgen**	-0.27	$7.9 * 10^{-1}$
**Estrogen**	-0.79	$4.3 * 10^{-1}$
**VEGF**	-1.58	$1.1 * 10^{-1}$
**P53**	-2.26	$2.4 * 10^{-2}$
**TGFB**	-2.91	$3.6 * 10^{-3}$

Supplementary Table S8. Single Cell Sequencing report

	Sequencing	Mapping*		Cells			Sequencing
**Sample**	**Total raw reads**	**to Genome**	**To Exons**	**Cell cnt**	**Reads/cell#**	**Genes/cell†**	**Sat. curve**
WT	148M	89.90%	73.60%	2,087	70,848	1,978	84% (OK)
PHD2cko	85M	87.60%	71.20%	1,444	58,954	1,595	84% (OK)

*, percentage of reads mapped confidently; cnt, count; #, mean; † median; Sat. saturation

SUPPLEMENTAL METHODS

ANIMALS

All mouse experiments were approved by the regulatory authority of the Maastricht University Medical Centre and performed in compliance with the Dutch governmental guidelines. Whole-body PHD1ko and PHD3ko mice,[54, 55] PHD2 conditional knockout mice (PHD2cko)[56] and LysMCre transgenics,[57] were previously described. All PHD lines were crossed to low density lipoprotein receptor knockout (LDLRko) mice, obtained from an in-house breeding colony, originating from Charles River (Wilmington, MA, USA) and refreshed every 10 generations to avoid genetic drift. Male LDLrko mice were used as control in all experiments involving PHD1ko and PHD3ko. LysMCre LDLrko mice (hereafter referred to as PHD2 WT) served as control in all experiments with PHD2cko mice. All mice were crossed back on LDLrko C57Bl6/J background at least nine times. Animals were housed in the laboratory animal facility of Maastricht University under standard conditions. Food and water were provided *ad libitum* during the entire experiment. All animals were housed in individually ventilated cages (GM500, Techniplast) in groups of up to 5 animals per cage, with bedding (corncob, Technilab-BMI) and cage enrichment. Cages were changed weekly, reducing handling of the mice to one handling per week during non-intervention periods.

ATHEROSCLEROSIS QUANTIFICATION AND IMMUNOHISTOCHEMISTRY

Mice were euthanized with a pentobarbital overdose (100 mg/kg i.p.) and blood was withdrawn via the right ventricle for flow cytometry, absolute white and red blood cell counts (Coulter Ac.T diff, Beckman Coulter) and total cholesterol analysis. Mice were perfused via the left cardiac ventricle with PBS containing sodium nitroprusside (0.1 mg/ml; Sigma-Aldrich, Seelze, Germany). Aortic arch, root and organs were subsequently excised and fixed in 1% paraformaldehyde overnight and paraffin-embedded.

Aortic roots and arches were serially sectioned (4µm) and stained with hematoxylin and eosin (H&E, Sigma) for plaque area and lipid core content quantification. Five consecutive H&E sections at 20 µm intervals were analyzed blindly using computerized morphometry (Leica QWin V3, Cambridge, UK) and the sum of the three valves averaged per mouse. Necrotic core was defined as a-cellular and a-nuclear plaque area containing cholesterol clefts, and shown as the percentage necrotic content of the total plaque area. Sections within this 100 µm interval were used for remaining immunohistochemical

stainings. Antigen retrieval was performed at pH 6 (Dako REAL target retrieval, Dako) (for MAC3, αSMA, and collagen type I), pH 9 (tris-EDTA, made in-house) (for platelet derived growth factor receptor beta (PDGFRβ)) or trypsin digestion (for CD31). Mouse atherosclerotic plaques were characterized for macrophage content (MAC3+ area/plaque area, BD Cat. No. 553322), smooth muscle cell content (αSMA+ area/ plaque area, αSMA Sigma F3777), PDGFRβ content (PDGFRβ+ area/ plaque area (C-terminus) (ab32570, clone Y92), and total collagen content (picrosirius red area/plaque area, analyzed with polarized light microscopy, Polyscience 09400). Additionally, adventitial microvessel density (CD31+ microvessels/adventitial area, BD Cat. No. 550274), HIF1α (Novus Biologicals, NB100-449), Ki67 (Abcam ab15580), and SPP1 (Abcam ab8448) were stained. Specific antigen-antibodies were visualized using appropriate secondary antibodies and diaminobenzamidin (DAB), unless specified otherwise. Fibrous cap thickness was measured in Sirius red stained sections of the brachiocephalic artery (at least three continuous sections) and aortic root (two sections per plaque), where necrotic core size was largest. In the section with largest necrotic core cap thickness was determined at the thinnest part, as the area between the outer edge of the cap and the necrotic core boundary.

In situ apoptosis was quantified on single cell level with TdT-mediated dUTP-X nick end labeling (TUNEL) technology (apoptotic cells/mm^2 plaque are; Roche, 11684795910). FITC-dUTP and anti-FITC-HRP labeled antibody binding were visualized using AEC positive substrate chromogen (Dako, K3461). Pictures were converted to pseudo fluorescent images using standard the deconvoluting option (hematoxylin and DAB) in FIJI software, and reassigning hematoxylin and DAB spectra to a blue and red pseudo fluorescent color, respectively.

HUMAN TISSUE COLLECTION

Human plaque tissue collections were used: Maastricht Pathology Tissue Collection (MPTC),[38] and Biobank of Karolinska Endarterectomies (BiKE) for analysis of protein and mRNA levels using immunohistochemistry, in situ hybridization (ISH), microarrays and western blot analysis. Tissue collection was in line with the Dutch Code for Proper Secondary use of Human Tissue and respective local Medical Ethical Committees. Human studies of BiKE are approved by the Ethical Committee of Stockholm and follow the guidelines of the Declaration of Helsinki.

MPTC samples of human carotid autopsy samples (n=38, mean age 72 years, 64% men), were used for immunohistochemistry and ISH. Sample represented the following stages of atherosclerosis (n=8-10 per group): intimal thickening, pathological intimal thickening, thick fibrous cap (stable) atheroma, and plaque with intraplaque hemorrhage.[38]

In the BiKE cohort, symptomatic and asymptomatic patients (n=127) undergoing carotid endarterectomy (CEA) were consecutively enrolled in the study and plaques collected for histology and RNA lysates. Transcriptome was analyzed by Affymetrix microarray (Dataset available from Gene Expression Omnibus with accession nr. GSE125771) and interrogated for target genes. Demographics, experimental procedures and array analysis of the BiKE study cohort have been extensively described previously.[58, 59]

HUMAN PLAQUE IMMUNOHISTOCHEMISTRY AND MULTISPECTRAL IMAGING

PHD1, 2 and 3 protein expression was assessed in human carotid autopsy samples. PHD1 expression was analyzed by fluorescent immunohistochemistry. After antigen retrieval (target retrieval DAKO), slides were incubated overnight with rabbit anti-human PHD1 (1:100 Novus Biologicals NB100-310), followed by biotin-conjugated donkey-anti-rabbit, streptavidin-horseradish peroxidase (HRP) and Cy3 labelled tyramide signal amplification reagent (Perkin Elmer). Nuclei were visualized with DAPI. Following antigen retrieval, PHD2 (Novus Biologicals NB100-2219) and CD68 (DAKO, M0814) were analyzed by non-fluorescent immunohistochemistry, followed by multispectral imaging to convert into pseudo-fluorescent images. Multispectral imaging (MSI) was performed to analyze human PHD1 and 2 expression, and PHD-CD68 co-localization. Spectral images were taken between 420-720 nm (10 nm interval) at a 10x (human) magnification using a Nuance spectral imaging system (Perkin Elmer/Caliper Life Sciences, Hopkinton, MA, USA) mounted on a Leica DM4000 B LED microscope, in case of immunofluorescence by using filters for N21 and A. Single stained sections, one chromogen/fluoro-chrome or counter stain only (DAB, Vector Red, Vector Blue, Dyelight 594, hematoxylin, DAPI), were used to create a spectral library. Computational decomposition of the individual image components was performed using the spectral library and NuanceTM 3.0.2 software. Pseudo-colors were assigned to unmixed images and composite images showing co-localization were generated with the Nuance 3.0 software. Stainings were performed on serial sections.

PHD3 IN SITU HYBRIDISATION

As accurate, reliable PHD3 antibodies are not available at this moment in time, we here use *in situ* hybridization to locate PHD3 expression in human plaques. *PHD3* mRNA expression was determined by *in situ* hybridization in FFPE, advanced human plaques derived from autopsy, as a specific PHD3 antibody is not available. All solutions used were treated with DEPC and autoclaved before use to eliminate RNAse activity. Sections were deparaffinized, rehydrated and treated with proteinase K (Invitrogen, 25530-049) to remove endogenous tissue nucleases. PHD3 mRNA was targeted (target sequence (TACATGGTGGGATCCTGCGGATATTTCCAGAGGGGAAATC

ATTCATAGCAGATGTGGAGCCCATTTTTGACAGACTCCTGTTCTTCTGGTCAGATCGTAGGAACC CACACGAAGTGCAGCCCTCTTACGCAACCAG) by a double-DIG labeled nucleotide probe (TGGTTGCGTAAGAGGGCT) in microRNA ISH Buffer (Exiqon). Hybridization was performed at 55ºC overnight. After washing off excessive probe with PBS the DIG-labeled probe was targeted by anti-DIG-AP (Roche, ref. 11093274910). Positive signal was visualized with NBT/BCIP AP detection tablets (ref. 11697471001). Cells were counterstained with Fast Red (ScyTek Laboratories, NFS500). Images were converted to pseudo-fluorescence in Fiji.

CELL CULTURE

Bone marrow was isolated, and cells were cultured for seven days in RPMI-1640 (Gibco with Glutamax, 2g/L glucose) supplemented with 10% FCS, 100U/ml Penicillin-Streptomycin, and 15% L929-conditioned medium to generate bone marrow-derived macrophages (BMDM). Macrophage conditioned medium was obtained by culturing differentiated BMDM for 24h in DMEM GlutaMAX (Gibco 31966) supplemented with 2% FCS, 100U/ml Penicillin-Streptomycin, and 15% L929-conditioned medium in either normal oxygen conditions or hypoxia (0.1% O_2).

Primary vascular smooth muscle cells (SMC) were isolated by enzymatic digestion (4h at 37°C in DMEM with 3 mg/ml collagenase (Sigma, Zwijndrecht, the Netherlands) and 1 mg/ml elastase (Sigma, Zwijndrecht, the Netherlands) from aortas of 5-10 C57/Bl6 mice (after removal of endothelium and adventitia) and cultured in DMEM (Gibco, 31966047) supplemented with 10% FCS and 100U/ml Penicillin-Streptomycin. NIH/3T3 were cultured in DMEM (Gibco, 31966047) supplemented with 10% FCS and 100u/ml Penicillin-Streptomycin. Before experiments with macrophage conditioned medium,

SMCs and NIH/3T3 were starved for 24-48 hours respectively using DMEM supplemented with 0.1% FCS.

For gene intervention, cells were incubated with transfection agent Viromer BLUE (Lipocalyx, VB-01LB-01) together with siRNA for HIF1 (5'-GUCACCACAGGACAGUACA-3'), BNIP3 (5'-ACCUUCUGAUGAAGAUUUGGAUC-3') and scramble control (5'-GCUUAACCCGUAUUGCCUA-3') in a concentration of 25nM for 8 hours.

PROLIFERATION ASSAY

Proliferation of SMC and 3T3 fibroblasts in response to macrophage conditioned medium (see above) was measured using CellTiter-glo luminescent cell viability assay (Promega, G7570) to determine ATP content of cells according to manufacturer's protocol. Primary SMC (2x104 cells) were isolated and seeded in a 96 wells plate and allowed to attach for 24 hours. After starvation for 24h in DMEM containing 0.5% FCS, BMDM-conditioned medium was added to the cells and incubated for 72 hours. Luminescence was measured on a luminometer (Victor3, PerkinElmer) and proliferation calculated as difference on ATP between T0 and T72hrs. Proliferation of PHD2 WT and cko BMDMs was also measured on an ACEA xCELLigence (Roche). BMDMs ($8x10^3$ cells) were seeded on a gold electrode implemented in a 96 wells plate. Impedance was measured hourly and used to quantify proliferation (slope of impedance increment over time) using RCTA software (version 1.2, Roche).

MIGRATION ASSAY

Migration stimulation in primary murine SMC by conditioned macrophage medium of WT and PHD2cko was measured on an ACEA xCELLigence (Roche). SMC were starved in DMEM containing 0.5% FCS for 24 hours. Upper chambers of ACEA CIM 16 plates (ACEA, 20131122) were coated with 10ug/ml collagen G (Biochrom, L7213) for one hour per side prior to start of the experiment. Subsequently, lower chambers were equilibrated for the respective conditioned mediums and controls. BMDM-conditioned medium contained a final concentration of 1% FCS and 15% LCM. SMC ($4x10^4$ cells) were then added to the upper chamber and migration was monitored for 24h (hourly measurements), using the slope of the impedance increment over time.

INTRACELLULAR COLLAGEN CONTENT

Intracellular collagen content was measured using CNA35-FITC (Kindly provided by prof. Reutelingsperger, Biochemistry department Maastricht) shown to bind to collagen type I, III and IV.[60] SMCs and 3T3s were starved in DMEM containing 0.1% FCS for 24 or 48 hours, respectively. SMCs and 3T3s were subsequently treated with conditioned medium of either PHD2 WT or conditional knockout macrophages with or without a collagen producing stimulus (TGF-β1) (5 ng/ml, Biolegend, 763102) for 72 hours. Cells were fixed in 2% PFA for 15 min and permeabilized using 0.1% Triton X-100 in PBS for 15 min. Subsequently, cells were stained for internal collagen content with CNA35-FITC (1µM) and nuclei were stained with Hoechst (15µg/ml). Samples were analyzed using the BD Pathway 855 High Content Bioimager. Data was processed with Attovision and BD Diva software.

COLLAGEN SECRETION

After serum starvation, SMCs and 3T3s were treated with conditioned medium of either WT or PHD2cko macrophages for 72 hours. Culture medium of SMCs and 3T3s was collected after 72 hours and analyzed using Sircol soluble collagen assay as described by the manufacturer (Biocolor, S1000). In comparable subsequent experiments, TGF-β1 was added to the conditioned medium or proteins were heat-inactivated (30 min, 85°C) prior to addition to 3T3 fibroblasts.

MATRIX METALLOPROTEASE ACTIVITY ASSAY

The functional activity of matrix metalloproteinase (MMPs) was determined using OmniMMP™ fluorogenic substrate (Enzo Life Science, BML-P126-0001). The Mca-Pro-Leu-Gly-Leu-Dpa-Ala-Arg-NH2 substrate incorporates a quencher (N-3-(2,4-dinitrophenyl)-L-a,β-diaminopropionyl) and a fluorescent side (7-methoxycoumarin-4-yl)acetyl). Upon MMP cleavage of the quencher fluorescence can be detected. A total of 0.14*106 BMDM (lysed in PBS containing 1% Trition-X100) was incubated with 180mM $CaCl_2$ 65µM OmniMMP substrate at 37°C and the fluorescence was detected at an interval of two minutes on a Spectromax (Ex 328nm, Em 393nm, Molecular Devices SPECTRAmax M2). The slope of fluorescence intensity increase was subsequently analyzed.

APOPTOSIS

BMDMs were stimulated with 50µM 7-ketocholesterol (Sigma, C2394) or 50µg/ml oxLDL (Isolated as described elsewhere)[61] for 24 hours to induce apoptosis. After stimulation, nuclei were stained with Hoechst (15µg/ml) and apoptotic cells with fluorescently labeled AnnexinxA5-FP488 (produced by Prof. Reutelingsperger, Maastricht) for 15 min. Samples were analyzed using a high-throughput, fluorescent reporter system, coupled to automated microscopy (BD Pathway 855 High Content Bioimager). Data was processed with Attovision and BD Diva software.

LIPID UPTAKE

BMDMs were given fluorescently labeled oxidized LDL (Topfluor, Avanti lipids 810255P-1mg) for 2 hours. Thereafter, nuclei were stained with Hoechst (15µg/ml). Samples were analyzed using a high-throughput, fluorescent reporter system, coupled to automated microscopy (BD Pathway 855 High Content Bioimager). Data was processed with Attovision and BD Diva software.

RNA SEQUENCING OF CULTURED CELLS

For RNA sequencing cells in vitro, RNA was isolated from triplicates of WT and PHD2cko BMDMs 24 hour after seeding, and from triplicate fibroblasts after 72 hours exposure to WT or PHD2cko conditioned medium. Bioanalyzer confirmed intact RNA (RNA Integrity number 10) for sequencing of 10µg RNA, on the NextSeq 500 system using v 2.5 chemistry, at ~15M single reads per sample by the c(Core Facility Genomics of the Medical Faculty Münster.

The gene-level expression of a total of 32,544 genes were quantified, 14,285 genes were retrieved for downstream analysis, 18,259 genes were discarded as lowly expressed genes.

BIOINFORMATICS ANALYSIS OF BULK RNA SEQUENCING DATA

Gene-level expression was quantified using Kallisto with the mouse genome (Mus Musculus GRCm38 assembly).[62] Principal component analysis was used for exploratory data analysis using the prcomp function in R (stats package, R 3.6.1 version). The limma R package (v3.40.6) was used to test for differential expression between conditions using the empirical Bayes method after voom transformation.[63] Lowly and non-expressed genes in the experiment were discarded from the analysis using the filterByExpr function (limma) to reduce potential false positives from the multiple testing.

Single-sample transcription factor activities were estimated using DoRothEA mouse regulons with A, B and C confidence classes.[64,21] Similar to differential gene expression analysis, the empirical Bayes method (limma) was also used to test for differential transcription factor activities using the TF activities (normalized enrichment scores) estimated by VIPER method (v.1.18.1). Genes and Transcription factor activities differentially dysregulated with FDR-adjusted p-values < 0.05 were considered significant. Pre-ranked Gene-Set Enrichment Analysis (GSEA) was performed using fgsea R package (v1.10.1) on the transcriptome-wide ranking of differential expression by the moderated t-statistics with the hallmark gene set collection from MSigDB and mouse gene sets from MatrisomeDB.[65, 66] Human genes from the hallmark gene set collection were transformed to their orthologs in mouse using the biomaRt service from EnsEMBL. Gene sets with FDR < 0.05 were considered significantly enriched in the condition. Pathway analysis was performed using PROGENy[67, 68] with the mouse model of pathway footprints of 100 genes, and 10,000 gene permutations of the ranking to build a null distribution for statistical estimations of significance. NicheNet [69] was used for the ligand-receptor analysis of stimulated pro-fibrotic fibroblasts by BMDM PHD2cko using the differentially over-expressed ligands from BMDMs PHD2cko (p-value < 0.05), and those target genes that were detected by the leading edge analysis in the significantly enriched MatrisomeDB gene sets from the pre-ranked GSEA in pro-fibrotic fibroblasts.

SINGLE CELL RNA SEQUENCING OF MURINE PLAQUES

PHD2 WT and PHD2 cko mice (n=11 and 9, respectively) were euthanized with a pentobarbital overdose (100 mg/kg i.p.) after 20 weeks of HCD and blood was withdrawn via the vena cava, followed by PBS perfusion via the left ventricle. The aortic root was subsequently excised and fixed in 4% paraformaldehyde overnight and paraffin-embedded.

Aortic arches of either genotype were pooled, sliced in pieces using a scalpel, and enzymatically digested for 15 minutes at 37°C using collagenase B (0.00284g/ml, Sigma 110088807001), pronase (0.01g/ml, Sigma 10165921001) and DNAse (0.1mg/ml, Roche 11284932001). Tissue was filtered through a 70µM strainer and subjected to red blood cell lysis (8.4g NH4CL + 0.84g NaHCO3 in 1 liter H2O, pH 7.2-7.4). All single, DAPI-negative living, cells were sorted on FACS Aria III for SCS. Cell count and viability of sorted cells was confirmed by trypan blue staining, and a total of ~12.000 DAPI-plaque cells were loaded on a chromium single cell controller using V2 reagent kit (10X Genomics) to

create cDNA sequencing libraries per manufacturers protocol. In short, in reaction vesicles (gel beads in emulsion, GEMs), cells were lysed and barcoded oligonucleotides reverse transcribed before clean-up and cDNA amplification. The Chromium Single-Cell 3' Library Kit was then used to generate indexed sequencing libraries. Sequencing was performed on a Novaseq 6000 system (Illumina) (Supplemental Table S7).

BIOINFORMATICS ANALYSIS OF SINGLE-CELL RNA-SEQUENCING DATA

Raw sequencing data (FastQ files) were processed (alignment and gene-level expression quantification) using the CellRanger pipeline (10x Genomics, version 3.1) with the mouse genome (mm10 assembly). Seurat R package (v.3.1.0) was used to perform a standard analysis.[70] Quality control diagnostics were applied on library sizes, percentage of mitochondrial genes and gene detection coverage of the single cells. Cells with a gene coverage between 500 and 4,000 of genes expressed, and less than 7.5% of mitochondrial gene expression were retrieved to avoid bad quality cells, such as doublets and dead cells in downstream analysis. After data normalization using log transformation and applying a 10,000 scaling factor, the 2,000 most highly variable genes were selected using the variance stabilizing transformation method implemented in Seurat for each sample. The first twenty principal components from Principal Component Analysis (PCA) applied on this selection of genes were used to find anchors for sample integration of the two conditions PHD2cko and WT pooled mice, and integrated using Canonical Correlation Analysis implemented in Seurat.[70] Myeloid leukocytes were identified following two rounds of unsupervised clustering. First, major clusters of cells expressing canonical myeloid leukocytes markers were selected. For this, PCA was applied on the scaled batch-corrected data to extract twenty-five principal components for the unsupervised clustering. Graph-based unsupervised clustering was performed using Shared-Nearest Neighbour algorithm. Louvain method was used to find clusters at resolution 0.1. Cluster of cells with positive expression of PTPRC, Lyz2, and CD68 expression, and absence of other vascular cell markers (CD3, CD19, MYH11, PECAM1) were detected as potential myeloid leukocytes. Second, this large and heterogeneous population of cells was selected for another iteration of unsupervised clustering to find more clusters with a higher resolution (at resolution 0.5), following the same workflow as described before. Distinct clusters were annotated based on the markers reported from Zernecke et al 2020, the top 10 marker genes for macrophages, monocytes, dendritic cells and top 10 over-expressed genes for Neutrophils as compared to the rest of cells.[53]. The main classes of myeloid

leukocytes were identified among clusters, including cavity, IFNIC, inflammatory, Trem2-foamy and resident macrophages, mature-DC, moDC, monocytes and neutrophils. In addition, a cluster of proliferating cells was detected using the CellCycleScoring function from Seurat with the ortholog genes in mouse of the cell cycling genes, and another small cluster remained as not assigned (n.a.) due to the non-specific expression of cell-type markers. Uniform Manifold Approximation and Projection (UMAP) was used to reduce the dimensional space of the twenty five principal components to an embedding of two dimensions for visualization purposes with standard parameters in the Seurat package. Pathway and transcription factor activities were estimated using PROGENy (https://saezlab.github.io/progeny/) and DoRothEA (https://saezlab.github.io/dorothea/) in single-cell data as previously described (pathway footprints of 100 genes and TF regulons of A, B and C confidence classes, respectively).[68] The enrichment of the PHD2cko signature expression in single-cell basis was calculated using AUCell R package (v1.6.1) using the top 50 most up-regulated genes in BMDM upon PHD2cko for the PHD2 functional stratification of plaque-resident macrophages, prior discarding the main molecular players Bnip3 and Spp1 as potentially being defined as part of the signature. Correlations analysis between Hif1a activity, hypoxia response and BMDM PHD2cko expression signature was performed using Pearson correlation. For differential gene expression analysis, the two clusters of proliferating and n.a. cells were leave out, and the two clusters of resident macrophages found in the unsupervised clustering were merged as a single population of cells. Differential gene expression was performed using Wilcoxon Rank Sum test. P-values from the Wilcoxon Rank Sum tests were adjusted for multiple testing using Bonferroni method for genome-wide multiple testing between two groups, and using FDR method for 2-group individual gene testing among cell types. Differentially expressed genes with adjusted p-values below 0.05 were considered statistically significant. R effect sizes from Wilcoxon Rank Sum test were calculated as Z divided by square root of the total observations. R effect size ranges from -1 to +1. Positive sign from r effect size relates to up-regulation in PHD2cko (group 1) as compared to WT condition (group 2). The greater the absolute r value is, the larger the effect size is. R effect size could be interpreted as small (r between 0.10 and 0.30), medium (r between 0.30 and 0.50) and large (r>0.50).

REFERENCES

53. Zernecke A, Winkels H, Cochain C, Williams JW, Wolf D, Soehnlein O, Robbins CS, Monaco C, Park I, McNamara CA, Binder CJ, Cybulsky MI, Scipione CA, Hedrick CC, Galkina EV, Kyaw T, Ghosheh Y, Dinh HQ, Ley K. Meta-Analysis of Leukocyte Diversity in Atherosclerotic Mouse Aortas. *Circ Res* 2020;**127**:402-426.

54. Aragones J, Schneider M, Van Geyte K, Fraisl P, Dresselaers T, Mazzone M, Dirkx R, Zacchigna S, Lemieux H, Jeoung NH, Lambrechts D, Bishop T, Lafuste P, Diez-Juan A, Harten SK, Van Noten P, De Bock K, Willam C, Tjwa M, Grosfeld A, Navet R, Moons L, Vandendriessche T, Deroose C, Wijeyekoon B, Nuyts J, Jordan B, Silasi-Mansat R, Lupu F, Dewerchin M, Pugh C, Salmon P, Mortelmans L, Gallez B, Gorus F, Buyse J, Sluse F, Harris RA, Gnaiger E, Hespel P, Van Hecke P, Schuit F, Van Veldhoven P, Ratcliffe P, Baes M, Maxwell P, Carmeliet P. Deficiency or inhibition of oxygen sensor Phd1 induces hypoxia tolerance by reprogramming basal metabolism. *Nat Genet* 2008;**40**:170-180.

55. Bishop T, Gallagher D, Pascual A, Lygate CA, de Bono JP, Nicholls LG, Ortega-Saenz P, Oster H, Wijeyekoon B, Sutherland AI, Grosfeld A, Aragones J, Schneider M, van Geyte K, Teixeira D, Diez-Juan A, Lopez-Barneo J, Channon KM, Maxwell PH, Pugh CW, Davies AM, Carmeliet P, Ratcliffe PJ. Abnormal sympathoadrenal development and systemic hypotension in PHD3-/- mice. *Mol Cell Biol* 2008;**28**:3386-3400.

56. Takeda Y, Costa S, Delamarre E, Roncal C, Leite de Oliveira R, Squadrito ML, Finisguerra V, Deschoemaeker S, Bruyere F, Wenes M, Hamm A, Serneels J, Magat J, Bhattacharyya T, Anisimov A, Jordan BF, Alitalo K, Maxwell P, Gallez B, Zhuang ZW, Saito Y, Simons M, De Palma M, Mazzone M. Macrophage skewing by Phd2 haplodeficiency prevents ischaemia by inducing arteriogenesis. *Nature* 2011;**479**:122-126.

57. Clausen BE, Burkhardt C, Reith W, Renkawitz R, Forster I. Conditional gene targeting in macrophages and granulocytes using LysMcre mice. *Transgenic Res* 1999;**8**:265-277.

58. Matic LP, Jesus Iglesias M, Vesterlund M, Lengquist M, Hong MG, Saieed S, Sanchez-Rivera L, Berg M, Razuvaev A, Kronqvist M, Lund K, Caidahl K, Gillgren P, Ponten F, Uhlen M, Schwenk JM, Hansson GK, Paulsson-Berne G, Fagman E, Roy J, Hultgren R, Bergstrom G, Lehtio J, Odeberg J, Hedin U. Novel Multiomics Profiling of Human Carotid Atherosclerotic

Plaques and Plasma Reveals Biliverdin Reductase B as a Marker of Intraplaque Hemorrhage. *JACC Basic Transl Sci* 2018;**3**:464-480.

59. Perisic L, Aldi S, Sun Y, Folkersen L, Razuvaev A, Roy J, Lengquist M, Akesson S, Wheelock CE, Maegdefessel L, Gabrielsen A, Odeberg J, Hansson GK, Paulsson-Berne G, Hedin U. Gene expression signatures, pathways and networks in carotid atherosclerosis. *J Intern Med* 2016;**279**:293-308.

60. Aper SJ, van Spreeuwel AC, van Turnhout MC, van der Linden AJ, Pieters PA, van der Zon NL, de la Rambelje SL, Bouten CV, Merkx M. Colorful protein-based fluorescent probes for collagen imaging. *PLoS One* 2014;**9**:e114983.

61. Redgrave TG, Roberts DC, West CE. Separation of plasma lipoproteins by density-gradient ultracentrifugation. *Anal Biochem* 1975;**65**:42-49.

62. Bray NL, Pimentel H, Melsted P, Pachter L. Near-optimal probabilistic RNA-seq quantification. *Nat Biotechnol* 2016;**34**:525-527.

63. Ritchie ME, Phipson B, Wu D, Hu Y, Law CW, Shi W, Smyth GK. limma powers differential expression analyses for RNA-sequencing and microarray studies. *Nucleic Acids Res* 2015;**43**:e47.

64. Garcia-Alonso L, Holland CH, Ibrahim MM, Turei D, Saez-Rodriguez J. Benchmark and integration of resources for the estimation of human transcription factor activities. *Genome Res* 2019;**29**:1363-1375.

65. Liberzon A, Subramanian A, Pinchback R, Thorvaldsdottir H, Tamayo P, Mesirov JP. Molecular signatures database (MSigDB) 3.0. *Bioinformatics* 2011;**27**:1739-1740.

66. Naba A, Clauser KR, Ding H, Whittaker CA, Carr SA, Hynes RO. The extracellular matrix: Tools and insights for the "omics" era. *Matrix Biol* 2016;**49**:10-24.

67. Holland CH, Szalai B, Saez-Rodriguez J. Transfer of regulatory knowledge from human to mouse for functional genomics analysis. *Biochim Biophys Acta Gene Regul Mech* 2020;**1863**:194431.

68. Holland CH, Tanevski J, Perales-Paton J, Gleixner J, Kumar MP, Mereu E, Joughin BA, Stegle O, Lauffenburger DA, Heyn H, Szalai B, Saez-Rodriguez J. Robustness and applicability of transcription factor and pathway analysis tools on single-cell RNA-seq data. *Genome Biol* 2020;**21**:36.

69. Browaeys R, Saelens W, Saeys Y. NicheNet: modeling intercellular communication by linking ligands to target genes. *Nat Methods* 2020;**17**:159-162.

70. Stuart T, Butler A, Hoffman P, Hafemeister C, Papalexi E, Mauck WM, 3rd, Hao Y, Stoeckius M, Smibert P, Satija R. Comprehensive Integration of Single-Cell Data. *Cell* 2019;**177**:1888-1902 e1821.
